# Microbiome‐urothelium crosstalk in bladder cancer: From dysbiosis to clinical translation

**DOI:** 10.1002/imo2.70081

**Published:** 2026-02-14

**Authors:** Haoxiang Xu, Zhibiao Li, Lin Yang, Jun Zheng, Weijia Li, Rulin Liao, Wenxue Huang, Boyuan Sun, Zhuohang Li, Cunzhen Ma, Xunguo Yang, Zongwei Wang, Jie Zhao, Bisheng Cheng, Peng Wu

**Affiliations:** ^1^ Department of Urology Nanfang Hospital, Southern Medical University Guangzhou China; ^2^ Department of Surgery, Division of Urology, Beth Israel Deaconess Medical Center Harvard Medical School Boston Massachusetts USA; ^3^ NMPA Key Laboratory for Research and Evaluation of Drug Metabolism, Guangdong Provincial Key Laboratory of New Drug Screening School of Pharmaceutical Sciences, Southern Medical University Guangzhou China; ^4^ Department of Clinical Oncology, Li Ka Shing Faculty of Medicine The University of Hong Kong Hong Kong SAR China; ^5^ Department of Clinical Oncology The University of Hong Kong‐Shenzhen Hospital Shenzhen China

**Keywords:** biomarker, bladder cancer, carcinogenesis, dysbiosis, immunotherapy, personalized medicine, urinary microbiome

## Abstract

Bladder cancer (BCa) ranks among the most frequently diagnosed malignancies worldwide. The advent of high‐throughput sequencing technologies has revealed distinct urinary microbial signatures that differentiate healthy individuals from BCa patients. While growing evidence implicates microbiota dysbiosis in the pathogenesis, progression, and therapeutic response of BCa, establishing a direct causal relationship remains challenging due to methodological heterogeneity across studies. This review summarizes the current knowledge of the human urinary microbiome (urobiome) in both health and disease states. It critically examines the evidence for microbiome involvement in BCa and discusses potential mechanisms, such as chronic inflammation, immune modulation, and genotoxin production, through which the microbiota may influence carcinogenesis. Furthermore, we explore the translational potential of urinary microbiota profiling as a biomarker for risk stratification and for predicting responses to intravesical therapies and immunotherapy in BCa patients. Unraveling the complexities of host–microbe interactions in BCa is essential for developing novel strategies for its prevention and personalized treatment.

## Highlights

- The urinary microbiome exhibits distinct dysbiosis in bladder cancer, providing specific microbial signatures for non‐invasive diagnosis and risk stratification.
- Mechanistic crosstalk drives tumorigenesis through chronic inflammation, bacterial genotoxins, and metabolic reprogramming via the gut–bladder axis.
- Microbial profiles serve as robust predictors of therapeutic efficacy for both intravesical Bacillus Calmette Guerin immunotherapy and systemic immune checkpoint inhibitors.
- Personalized strategies, including microbiome‐conscious antibiotic stewardship and probiotic interventions, offer novel avenues to enhance bladder cancer treatment outcomes.

## Graphical abstract

This review elucidates the critical crosstalk between the urobiome and bladder cancer (BCa), mapping the landscape from ecological dysbiosis to clinical translation. We synthesize emerging evidence on microbial signatures that distinguish BCa patients, exploring key carcinogenic mechanisms including chronic inflammation, genotoxicity, and the gut–bladder metabolic axis. Crucially, the review underscores the translational utility of the microbiome as a non‐invasive liquid biopsy for risk stratification and for predicting therapeutic responses to Bacillus Calmette Guerin immunotherapy and immune checkpoint inhibitors. Furthermore, we propose a forward‐looking framework for precision oncology, integrating microbiome‐based interventions—such as probiotics, targeted bacteriophages, and antibiotic stewardship—to modulate the tumor microenvironment and optimize patient outcomes.

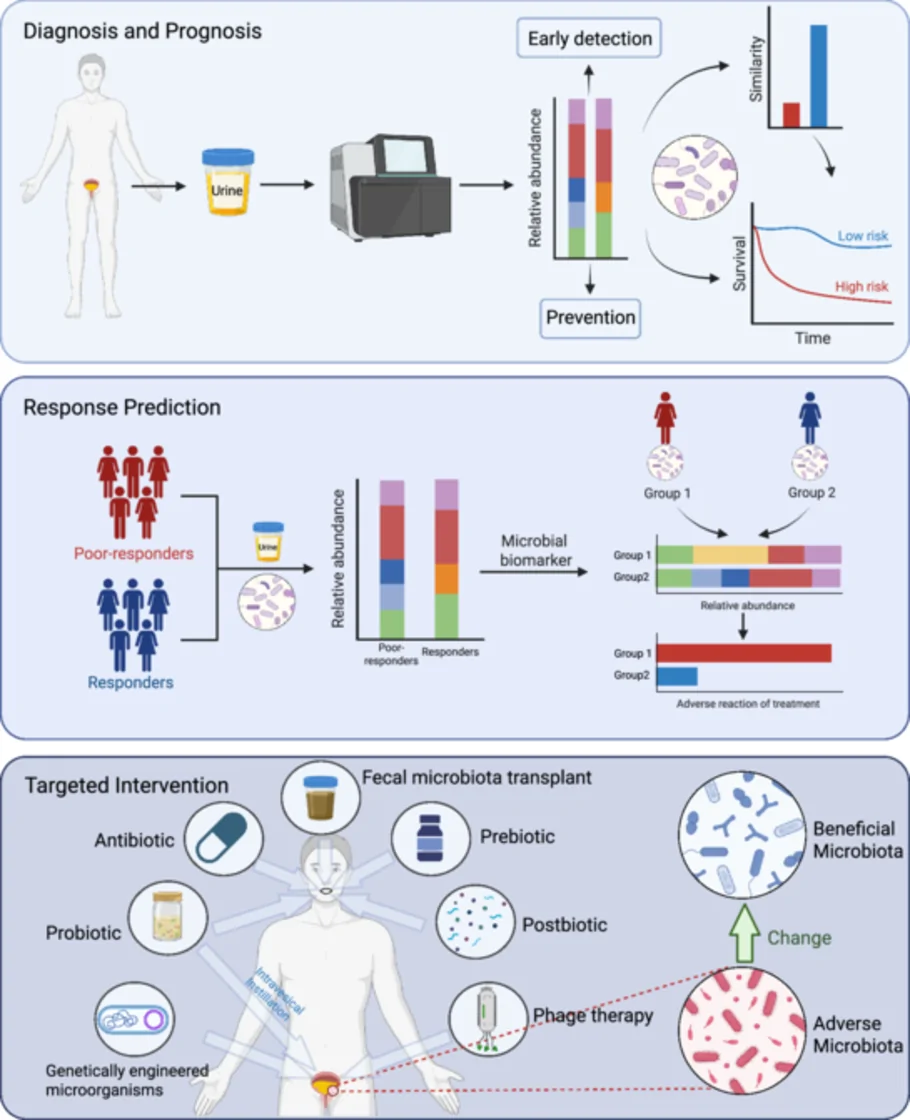

## 1 INTRODUCTION

Bladder cancer (BCa) is one of the most common genitourinary malignancies, characterized by high incidence rates and significant impacts on morbidity and mortality [1, 2, 3, 4]. Epidemiological reports from 2022 indicate more than 600,000 new cases and over 200,000 deaths worldwide, with a notably higher prevalence in males compared to females [2]. The incidence of BCa has risen in recent years, a trend ascribed to factors such as environmental pollution, population expansion, aging, and lifestyle choices—especially the increasing prevalence of smoking, a well‐established risk factor [3].

Clinical management and classification of BCa depend heavily on the degree of invasion, typically categorized as either non‐muscle invasive bladder cancer (NMIBC) or muscle invasive bladder cancer (MIBC). At initial diagnosis, NMIBC accounts for 70%−75% of cases, while that of MIBC is 20%–25%, and metastatic disease is present in approximately 5% of patients at the initial diagnosis [5, 6, 7, 8]. Histologically, urothelial carcinoma accounts for around 95% of BC cases, with other types, including squamous cell carcinoma, adenocarcinoma, and neuroendocrine carcinoma, constituting the remainder [6, 8]. The initial treatment for NMIBC involves transurethral resection of the bladder tumor (TURBT), often followed by intravesical chemotherapy or immunotherapy depending on a risk‐stratified approach to reduce recurrence and progression. Management of MIBC often involves radical cystectomy and urinary diversion, with adjuvant therapy utilized as indicated [7, 9, 10, 11]. Cystoscopy and urine cytology serve as fundamental diagnostic techniques, augmented by magnetic resonance imaging (MRI) for staging; however, both approaches have limitations. Urine cytology and MRI demonstrate limited sensitivity, while cystoscopy is invasive, costly, and linked to patient discomfort [12, 13, 14, 15]. Furthermore, despite progress in surgical techniques and perioperative chemotherapy, recurrence rates remain high: NMIBC exhibits a 5‐year recurrence rate ranging from 31% to 78%, and almost 30% of patients receiving cystectomy experience recurrence within a median of 12 months [9]. Moreover, long‐term survival outcomes have shown no improvement in recent decades, highlighting the pressing need for innovative approaches to improve diagnostic and therapeutic effectiveness.

Parallel to these clinical challenges, the role of the microbiome in oncology has gained increasing prominence, with associations dating back as far as 1813 when Vautier recorded the delayed growth of tumors in cancer patients suffering from gas gangrene [16, 17]. However, the technological constraints of the era hindered the discovery of relations between microbiome and cancer. A pivotal advancement came in 1974, when Professor Harald zur Hausen established the link between human papillomavirus (HPV) and cervical cancer [18]—a landmark discovery that provided the first definitive evidence of a specific microbe driving human oncogenesis. This was further solidified in 1983, when Marshall BJ and Warren JR successfully cultured Helicobacter pylori and elucidated its causal role in gastric carcinogenesis [19], underscoring the importance of microbes in cancer development.

With the advancement of biotechnology (including sequencing methods, imaging techniques, and culturomics, etc.), a large number of studies have revealed the mechanisms by which the microbiota influences tumor progression, metastasis, and treatment outcomes. For instance, Roje et al. reported that the gut microbiota might promote the progression of BCa in mice exposed to N‐butyl‐N‐(4‐hydroxybutyl)‐nitrosamine (BBN) [20]. Chen et al. conducted in vivo experiments in a melanoma mouse model, validating that intratumoral injection of *Burkholderia cepacia*, *Priestia megaterium*, and *Corynebacterium kroppenstedtii* acts synergistically with anti‐programmed cell death protein 1 (anti‐PD‐1) treatment to inhibit tumor growth and enhance anti‐tumor immunity [21]. Jin et al. successfully identified 56 novel bile acids derived from the intestinal microbiota and found that one type of bile acid could exert anti‐tumor immunomodulatory effects in the host by antagonizing androgen receptor (AR) in CD8 + T cells [22].

The emergence of microbiome research in oncology marks a paradigm shift in cancer biology, transitioning from a cell‐centric view to a holistic understanding that incorporates the critical influence of commensal microorganisms. This rapidly expanding field has established that the microbiome—particularly the gut and tumor‐resident microbiota—plays a fundamental role in the etiopathogenesis of various malignancies.

While the gut microbiome remains a primary focus of research, growing attention is also being directed toward the urinary microbiome (urobiome), which encompasses the diverse microbial communities residing within the urinary tract. In recent years, in‐depth research into the microbiota has revealed that many tissues, including the lungs, prostate, bladder, breasts, liver, and pancreas, which were previously considered sterile, actually contain microorganisms and are closely related to various physiological and pathological processes [23, 24, 25, 26, 27, 28]. Furthermore, the long‐standing belief in urine sterility has been overturned. Evidence confirms that the urinary tract possesses its own resident microbiota, which influences a range of urologic conditions, including overactive bladder, interstitial cystitis, and neurogenic bladder [29, 30, 31].

More recently, compelling evidence has emerged linking dysbiosis of the urobiome to BCa, suggesting its potential role as both a biomarker and a therapeutic target [32]. Studying the urobiome may not only reveal novel mechanisms of carcinogenesis and progression, such as chronic inflammation and immune modulation, genotoxin‐induced DNA damage by pathogenic bacteria, and metabolic reprogramming that activates tumor‐promoting pathways, but also support the development of non‐invasive diagnostic tools based on microbial signatures. Furthermore, it opens the door to innovative microbiota‐based therapeutic strategies, including probiotics, engineered bacteria, or antibiotics tailored to restore a healthy urobiome. Therefore, this review examines the new frontiers of microbial signatures in BCa concerning diagnosis, prognosis, and therapy, aiming to offer innovative insights for the diagnosis and treatment of BCa.

## 2 URINARY MICROBIOME: COMPOSITION AND CHARACTERISTICS

### 2.1 Overview of normal urinary microbiome

Historically, standard urine culture techniques were the primary method for studying urinary bacteria; however, these methods are limited in their ability to detect anaerobic or slow‐growing bacteria, leading to the long‐held misconception that urine is sterile. Recent advances in molecular techniques, such as 16S rRNA amplicon sequencing, whole‐genome shotgun metagenomic sequencing (WGMS), and enhanced quantitative urine culture (EQUC), have revealed that urine from healthy individuals harbors a diverse resident bacterial community [33].

Large‐scale studies have helped characterize this community. One study employing whole metagenomic and whole‐genome sequencing of urine samples from 1579 healthy young adults found the most abundant genera to be, in descending order: *Lactobacillus*, *Variovorax*, *Acinetobacter*, *Prevotella*, and *Sphingobium*. At the species level, *Lactobacillus iners* (*L. iners*) had the highest relative abundance, followed by *Variovorax* sp. PDC80, *Sphingobium fluviale*, *Lactobacillus crispatus* (*L. crispatus*), and *Gardnerella vaginalis* (*G. vaginalis*) [34]. Another study performed 16S rRNA gene sequencing on midstream urine specimens from 1172 healthy participants aged 45 to 86, reported the top six most abundant genera as *Prevotella*, *Streptococcus*, *Lactobacillus*, *Gardnerella*, *Escherichia‐Shigella*, and *Veillonella* [35]. Furthermore, several genera, including *Lactobacillus*, *Corynebacterium*, *Prevotella*, *Staphylococcus*, *Streptococcus*, *Escherichia*, *Enterococcus*, and *Citrobacter* are commonly detected in both men and women, while *Pseudomonas* appears to be exclusively identified in men [36, 37].

To date, few studies have focused specifically on defining the “healthy” urobiome, and in most research, healthy individuals serve as controls in studies of urinary system diseases. Nonetheless, *Lactobacillus* and *Streptococcus* are consistently among the most frequently reported genera in the urobiome of healthy populations [30].

### 2.2 Factors influencing microbiome composition (e.g., sex, age, and antibiotics)

The composition of the microbiome in a healthy human body is believed to be influenced by multiple factors, including gender, age, and antibiotics use. It is generally accepted that the composition of the urine microbiome differs between male and female patients. However, a study that utilized 16S rRNA sequencing to characterize the urobiomes of 20 healthy individuals (10 males and 10 females) found no differences at the phylum level between men and women. The urobiome is predominantly composed of four major phyla, including the phylum *Firmicutes* (65% in males vs. 73% in females), *Actinobacteria* (15% males vs. 19% females), *Bacteroidetes* (10% males vs. 3% females), and *Proteobacteria* (8% males vs. 3% females) [36]. Conversely, statistically significant disparities emerged between males and females at the bacterial genus level; the males exhibited a higher abundance of *Streptococcus* (31%), whereas females were dominated by *Lactobacillus* (63%). The current consensus indicates that the male urobiome is predominantly characterized by *Corynebacterium* and *Streptococcus*, while the female urobiome is marked by higher abundances of *Lactobacillus* and *Gardnerella* [36, 38, 39, 40]. In addition, Urine samples from premenopausal women typically exhibit a urotype dominated by *Lactobacillus*, whereas postmenopausal women display a more diverse urinary microbiota with increased abundance of *Gardnerella* and *Prevotella* [41]. *Lactobacillus*, a facultative anaerobic and acidogenic bacterium, plays a protective role in the vaginal environment by reducing pH and secreting antimicrobial compounds [42].

Notably, gender‐related factors, including sex hormones, were identified as key determinants of urobiome composition. For example, *Variovorax* sp. PDC80 and *Acinetobacter junii*, which are more prevalent in males, are associated with elevated serum testosterone, urine‐specific gravity, muscle mass, basal metabolic rate, serum creatinine, and serum uric acid‐parameters typically higher in males. In contrast, *G. vaginalis* and *L. iners*, more frequently observed in females, correlate significantly with higher serum estradiol levels. The urobiome also demonstrated strong gender‐predictive capability, achieving an area under the curve (AUC) of 0.843 [34].

The relationship between sex and urinary microbial diversity remains contested, with studies reporting conflicting results. The variation of the urine microbiome can be characterized by α‐diversity (the diversity within a sample) and β‐diversity (the differences between samples) [40]. One large‐scale study (*n* = 1165) employing 16S rRNA sequencing of midstream urine suggests that the male urobiome exhibits higher α‐diversity than that of females [35], a difference attributed to the greater abundance of lactic acid bacteria in females [43]. In direct contrast, another large population cohort (*n* = 1579) utilizing whole‐genome shotgun (WGS) metagenomic sequencing observed significantly higher α‐diversity in the female urinary microbiota [34]. This discrepancy likely reflects the higher resolution of WGS, which captures a broader spectrum of microorganisms (including fungi and viruses) and avoids the primer bias inherent to 16S amplicon sequencing. Further complicating the picture, a separate exploratory study (*n* = 42) using 16S rRNA sequencing on mixed sample types found no significant differences in diversity [44]. These disparate findings highlight that both sampling method and sequencing platform (16S rRNA gene amplicon sequencing vs. WGS) are critical determinants of study outcomes.

Age has rarely been investigated as a factor influencing changes in the urobiome. In an early pilot study (*n* = 26), Lewis et al. investigated the urobiome of healthy individuals aged 26 to 90 years using 16S rRNA gene amplification, after categorizing the samples into age groups (20−49, 50−69, and ≥70 years), they found that *Jonquetella*, *Parvimonas*, *Proteiniphilum*, and *Saccharofermentans* were unique to individuals over 70 years old [45]. However, findings from recent large‐scale population cohorts offer more robust insights. Zou et al. analyzed 1579 younger adults (mean age 29 years) and found the urobiome to be predominantly composed of *Lactobacillus* and *Variovorax* [34], whereas Qin et al. characterized 1172 elderly individuals (mean age 65.6 years) and identified *Prevotella* and *Streptococcus* as the dominant genera [35]. Notably, the abundance of *Lactobacillus* was significantly lower in the elderly cohort [46, 47]. Additionally, an *Escherichia*‐dominated urotype has been associated with healthy elderly women, whereas a *Gardnerella*‐dominated urotype is more common in young women [48]. These age‐ and sex‐related variations in the urobiome are to be expected and can be attributed to differences in urinary metabolites, voiding habits, and hygiene practices, as well as anatomical, hormonal, and histological differences between men and women.

Antibiotic use also influences the composition of the urinary microbiota. Antibiotics commonly prescribed for urinary tract infections (UTIs) not only impact the gut microbiome but also reduce the diversity and abundance of commensal bacteria in the urinary tract [49, 50, 51, 52]. Although studies on the effects of antibiotics on the urinary microbiota remain limited, existing evidence indicates that they contribute considerably to its compositional variation [53]. For instance, Gottschick et al. reported in a longitudinal cohort of 27 patients that metronidazole treatment led to a twofold increase in the relative abundance of *L. iners*, while significantly reducing the Shannon diversity and the mean relative abundance of *G. vaginalis*, *Atopobium vaginae*, and *Sneathia amnii* [53]. Similarly, in a larger cross‐sectional study of 148 individuals, Mulder et al. observed a subsequent rise in the abundance of *Escherichia coli* (*E. coli*) and *Parabacteroides* in voided urine samples, accompanied by a decrease in *Finegoldia* and *Lactobacillus* [54]. Additionally, recent research on tissue microbiomes (*n* = 34) has shown that the use of antibiotics in patients with BCa can reduce the abundance of *Bacteroides* and increase the abundance of *Staphylococcus* in tumor tissues [55]. However, a major limitation of these studies is the reliance on voided urine in some cohorts and the lack of long‐term follow‐up to determine if the microbiome fully recovers to its pre‐antibiotic state.

### 2.3 Sampling methods and limitations in urinary microbiome studies

Urinary microbiome research faces unique challenges, primarily due to the low biomass of urine samples, which increases the risk of contamination and technical biases. Addressing these issues requires stringent optimization across all stages‐including sample collection, processing, and bioinformatic analysis. The method of urine collection is particularly critical in study design, as different sampling techniques may introduce distinct types of biological contaminants (Figure 1A). Common methods include midstream clean‐catch urine (CC), intermittent or permanent transurethral catheter (TUC), and suprapubic aspiration (SPA) [56].

**FIGURE 1:**
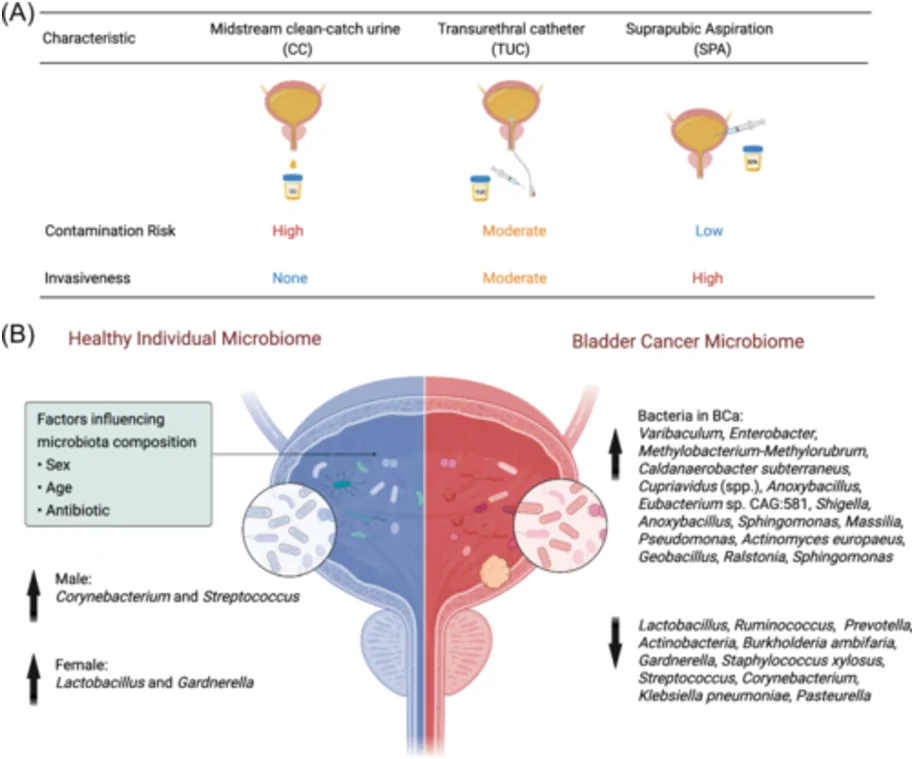
Comparison of the urinary microbiome in health versus cancer and implications of sampling methodologies. (A) Evaluation of urine sampling techniques. The accuracy of microbiome profiling is critically dependent on the sampling method. Midstream clean‐catch urine (CC), while non‐invasive, carries the highest risk of contamination from genital skin microbiota (pollution risk). In contrast, Transurethral catheterization (TUC) and Suprapubic aspiration (SPA) provide more representative bladder‐specific samples but with increasing degrees of invasiveness. (B) The transition from homeostasis to dysbiosis. The left panel depicts the healthy microbiome, while the right panel illustrates the bladder cancer (BCa) microbiome. Upward arrows indicate genera enriched in BCa; downward arrows indicate those decreased.

CC urine is collected after its passage through the entire lower urinary tract. A major advantage of this method is its minimal invasiveness, reducing potential harm and discomfort to the patient. However, without prior disinfection of the periurethral area, this method is susceptible to contamination from microbial communities of the urethra, genitalia, and skin [57, 58]. Compared to CC, TUC offers more specific sampling of urinary microbial communities. Nevertheless, insertion of the catheter through the urethra may disrupt and co‐sample urethral microbiota [58, 59]. One technique to differentiate between disturbed urethral microbes and true bladder‐resident microbiota is the concurrent collection of urethral swabs for contamination assessment. SPA remains the most accurate method for exclusive sampling of bladder‐resident microbiota [60, 61]. However, it is also the most invasive approach and is difficult to ethically justify, particularly in elderly populations [59]. During SPA, a needle is inserted through the abdominal wall above the pubic bone into the bladder, allowing urine to be collected directly [62]. Indeed, some studies suggested that SPA is optimal for minimizing vulvovaginal and urethral contamination when studying the urobiome, compared to TUC [59, 61]. Nonetheless, some reports indicate that TUC can yield samples comparable to those obtained via SPA [63, 64]. Balancing the advantages and limitations of these sampling techniques is a critical step in selecting the most appropriate method for future research. In addition, recent consensus recommends using distinct terminology to differentiate sample types: samples collected directly from the bladder (via TUC, SPA, or cystoscopy) should be termed “urinary bladder,” whereas CC samples are more appropriately labeled “urogenital” because of the higher risk of contamination. This distinction supports more precise classification and investigation of urinary tract microbiota [65].

The urinary microbiome is characterized by its low biomass, typically with bacterial densities below 10^5^ colony‐forming units per milliliter [60]. In contrast, bacterial niches such as the gut and vagina can reach densities as high as 10^11^ bacteria per gram [66]. DNA contamination poses a major challenge in urinary microbiome studies because of the low biomass, which may lead to an overestimation of certain taxa [67]. So, stringent precautions during sample collection, processing, and bioinformatic analysis are essential to minimize contamination.

To mitigate the confounding effects of sampling heterogeneity, this review adheres to the standardized nomenclature established by the UROBIOME consensus statement [65]. We distinguish the “Urogenital Microbiome”—derived from voided urine—as a composite community containing bladder, urethral, and genital flora. In contrast, we use the term “Urinary Bladder Microbiome” strictly for samples obtained via transurethral catheterization or SPA, which bypass the distal urethra to reflect the bladder lumen environment. Finally, the “Urothelial (or Tissue) Microbiome” refers to microbiota detected in biopsies, representing the specific niche of adherent or intracellular bacteria distinct from the planktonic communities found in urine.

## 3 DYSBIOSIS IN BCA: ASSOCIATIONS AND MECHANISMS

### 3.1 From homeostasis to dysbiosis: Shifts in microbial diversity and composition

The transition from health to disease in the bladder is marked by profound alterations in microbial ecology. While healthy urine is typically homeostatic, BCa patients exhibit distinct dysbiosis manifested through changes in microbial diversity, richness, and the differential abundance of specific bacterial taxa (Figure 1B).

Current evidence regarding microbial diversity and richness in BCa remains heterogeneous, likely reflecting variations in sample types, methodologies, and cohort characteristics. Several studies report significant reductions in diversity and richness. For instance, an investigation involving 34 BCa patients and 29 controls found reduced microbial richness and diversity in aseptic catheterized urine samples from cancer cases (Taxonomic unit counts: 83 vs. 139, *p* = 0.015; Shannon index: 2.46 vs. 2.94, *p* = 0.049) [68]. Similarly, a prospective study of 27 BCa patients observed higher microbial richness and evenness in voided urine samples from controls compared to patients [69], and BCa tissue samples have historically displayed diminished microbial richness compared to normal adjacent tissues [70].

Conversely, other studies have reported increased microbial richness in BCa cohorts. A study of 62 male patients demonstrated significantly higher richness indices (Observed Species, Chao1, and Ace; all *p* < 0.01) in the urine of the cancer group compared to controls [71], with receiver operating characteristic (ROC) analysis suggesting richness could serve as a diagnostic marker. This pattern was echoed in an analysis of the urogenital microbiome (clean‐catch midstream urine) of 31 BCa patients, which reported significantly increased bacterial richness in the tumor group [72]. Adding to the complexity, several studies found no statistically significant differences in diversity metrics [73, 74, 75, 76, 77], underscoring the substantial variability inherent in current microbiome research.

Despite the inconsistencies in diversity metrics, specific taxonomic shifts frequently distinguish BCa patients from healthy controls. Detailed comparisons of enriched and depleted taxa across urine and tissue samples are comprehensively summarized in Table 1. In urine, 16S rRNA sequencing of aseptic catheterized samples revealed that genera such as *Veillonella*, *Varibaculum*, and *Methylobacterium‐Methylorubrum* were enriched in the cancer group, whereas *Pasteurella*, *Corynebacterium*, and *Acinetobacter* were more abundant in controls [68]. More comprehensive RNA‐sequencing analyses of urine samples (*n* = 18 vs. 12, validated in *n* = 81) confirmed a consistent depletion of *Escherichia* and *Acinetobacter* alongside an enrichment of *Enterobacter* in cancer samples [87].

**TABLE 1:** Summary of microbial dysbiosis and potential biomarkers in bladder cancer.

Biomarker source	Microbes (Genus/Species)	Sample type	Changes in BCa patients	Clinical significance and association	Level of evidence	Representative references
Urine‐Based	*Veillonella*	Urine	Enriched/Decreased	Diagnosis: Frequently enriched in BCa patient urine	Clinical Association	[68, 77, 78]
Urine‐Based	*Varibaculum*, *Methylobacterium‐Methylorubrum*	Urine	Enriched	Diagnosis: Enriched in BCa urine	Clinical Association	[68]
Urine‐Based	*Enterobacter*	Urine	Enriched	Diagnosis: Enriched in BCa urine	Clinical Association	[69, 73]
Urine‐Based	*Caldanaerobacter subterraneus*	Urine	Enriched	Diagnosis: Enriched in BCa urine	Clinical Association	[69]
Urine‐Based	*Acinetobacter*	Urine	Enriched/Decreased	Diagnosis: Inconsistent findings, which are likely dependent on the sample type. Prognosis: Enriched in the urine of the recurrence group	Clinical Association	[68, 69, 76, 79]
Urine‐Based	*Cupriavidus* (spp.)	Urine	‐	Prognosis: Enriched in the urine of the recurrence group	Clinical Association	[80]
Urine‐Based	*Anoxybacillus*	Urine	‐	Prognosis: Enriched in the urine of the recurrence group	Clinical Association	[71]
Urine‐Based	*Escherichia‐Shigella*	Urine	Decreased	Diagnosis: Decreased in BCa urine; BCG Response: Enriched in urine of BCG non‐responders; Mechanism of Escherichia: Genotoxin‐producing (pks+ *E. coli*) strains drive tumorigenesis.	Preclinical/Clinical Association	[69, 81]
Urine‐Based	*Eubacterium* sp. CAG:581	Urine	Enriched	NMIBC Diagnosis and Prognosis: 3‐year prediction (AUC = 0.79) Mechanism: Drives tumor progression through the ECM1/MMP9 pathway.	Preclinical/Clinical Association	[82]
Urine‐Based	*Corynebacterium*	Urine	Enriched/Decreased	Diagnosis: Inconsistent (reported as enriched in both BCa patients and the non‐BCa controls). Prognosis: Inconsistent (enriched in the urine of recurrent patients at TURBT or postoperatively, but enriched in urine from non‐recurrent patients in other studies).	Clinical Association	[68, 77, 79, 80, 83]
Urine‐Based	*Lactobacillus	Urine	Decreased	Diagnosis: Decreased in BCa urine Protective role: association with a positive history of BCG therapy	Clinical Association	[73, 75, 78]
Urine‐Based	*Sphingomonas*	Urine	‐	Prognosis: Enriched in the urine of the non‐recurrent group	Clinical Association	[80]
Urine‐Based	*Massilia*	Urine	‐	Prognosis: Inconsistent (enriched in the urine of recurrent patients, while enriched in urine from non‐recurrent patients in other studies)	Clinical Association	[71, 80]
Urine‐Based	*Aeromonas*	Urine	‐	Prognosis: Enriched in the urine of the recurrent group	Clinical Association	[80]
Urine‐Based	*Pseudomonas*	Urine	Enriched	Prognosis: Inconsistent (enriched in the urine of recurrent patients, while enriched in urine samples at TURBT from non‐recurrent patients in other studies)	Clinical Association	[79, 83]
Urine‐Based	*Actinomyces europaeus*	Urine	Enriched	Diagnosis: Enriched in BCa Urine	Clinical Association	[78]
Urine‐Based	*Geobacillus	Urine	Enriched	Diagnosis: Enriched in BCa Urine	Clinical Association	[76]
Urine‐Based	*Ralstonia*	Urine	‐	Prognosis: Enriched in the urine of the recurrent group	Clinical Association	[80]
Urine‐Based	*Ruminococcus	Urine	Decreased	Diagnosis: Decreased in BCa urine Prognosis: Enriched in the urine of the non‐recurrent group	Clinical Association	[77, 83]
Urine‐Based	*Aerococcus*	Urine	‐	BCG Response: Enriched in urine of BCG non‐responders;	Clinical Association	[81]
Urine‐Based	*Bifidobacterium breve/longum*	Urine	‐	BCG Response: Enriched in urine of BCG responders;	Clinical Association	[84]
Tissue‐Based	*Actinobacteria*, *Prevotella*, *Lactobacillus, *Ruminococcus	Tissue	Decreased	Diagnosis: Decreased in BCa tissue	Clinical Association	[70]
Tissue‐Based	*Acinetobacter*, *Escherichia‐Shigella*, *Cupriavidus* (spp.), *Anoxybacillus, *Geobacillus, *Sphingomonas*	Tissue	Enriched	Diagnosis: Enriched in BCa tissue	Clinical Association	[70]
Tissue‐Based	*Corynebacterium*	Tissue	‐	BCG Response: Inconsistent (reported as enriched in tissues of both BCG responders and non‐responders).	Clinical Association	[85, 86]
Tissue‐Based	*Ralstonia*	Tissue	Enriched	Diagnosis: Enriched in BCa tissues Prognosis: Enriched in the urine of the recurrent group	Clinical Association	[70, 80]

*Note*: * indicates microbial genera that exhibit consistent enrichment or depletion trends in both urine and tissue samples, suggesting their potential as robust diagnostic biomarkers.

Abbreviations: AUC, area under the curve; BCG, Bacillus Calmette‐Guérin; BCa, bladder cancer; ECM1, extracellular matrix protein 1; *E. coli*, *Escherichia coli*; MMP9, matrix metalloproteinase 9; NMIBC, non‐muscle invasive bladder cancer; TURBT, transurethral resection of bladder tumor.

In bladder tissues, significant ecological dysbiosis is also evident. Beneficial genera such as *Lactobacillus*, *Prevotella*_9, and *Ruminococcaceae* are generally reduced in tumor tissues, while genera including *Cupriavidus* spp., *Acinetobacter*, *Anoxybacillus*, *Escherichia‐Shigella*, and *Sphingomonas* are notably enriched [70]. A separate study of bladder tumor tissue from 32 patients confirmed these microbiota alterations, finding that *Actinobacteria* were significantly more enriched in non‐tumor mucosa and that microbial composition differed according to tumor grade [88]. While some studies suggest consistency between urinary and tissue microbiomes [75, 89], others have identified discrepancies, noting that genera like *Akkermansia*, *Bacteroides*, and *Klebsiella* may be significantly more abundant in tissue samples than in urine.

Furthermore, other studies on the urobiome in BCa are also predominantly small‐scale and exhibit considerable heterogeneity in their findings and conclusions [72, 73, 74, 78]. These heterogeneous outcomes may be attributed to factors and sampling methodologies. Gender is a major determinant; a large‐scale analysis revealed sex‐specific alterations where *Klebsiella* was enriched in the urine of female BCa patients, whereas no clinically relevant genera were differentially enriched in males [75]. Smoking, a well‐established risk factor, also shapes the microbiome, with smokers exhibiting significantly higher abundances of *Bacteroidaceae*, *Erysipelotrichales*, *Lachnospiraceae*, and *Bacteroides* compared to non‐smokers [76]. Furthermore, the method of urine collection significantly biases composition: compared to controls, catheter‐collected urine from BCa patients showed increased enrichment of *Veillonella* and *Corynebacterium*, whereas midstream urine samples demonstrated significant increases in *Streptococcus*, *Enterococcus*, and *Fusobacterium* [77].

### 3.2 Inflammation and immune modulation: How dysbiosis shapes the tumor microenvironment

It is well‐established that inflammation, whether local or systemic, plays a pivotal role in Carcinogenesis. Infections trigger acute inflammatory reactions that can progress into chronic inflammation due to persistent activation of host defense mechanisms against microbial invasion or tissue damage, thereby establishing a microenvironment that supports tumor development [90] (Figure 2A). A central mechanism underlying this progression is dysbiosis, defined as microbial imbalance with increased opportunistic pathogens, which perpetuates antigenic stimulation and sustains chronic inflammation [91]. Clinically, this is exemplified by recurrent urinary tract infections, a recognized risk factor for BCa [92]. Similarly, chronic *Schistosoma haematobium* infection promotes bladder carcinogenesis via the generation of carcinogenic N‐nitrosamines and the perpetuation of a pro‐tumorigenic inflammatory environment [93, 94]. Sustained inflammatory activity damages the urothelial barrier through processes including continuous generation of reactive oxygen and nitrogen species (ROS/RNS), leading to epithelial cell death, loss of barrier integrity, and further tissue injury [95, 96]. Notably, ROS/RNS induce DNA lesions such as 8‐oxoguanine and promote genomic instability, directly contributing to mutations in oncogenes and tumor suppressor genes [97].

**FIGURE 2:**
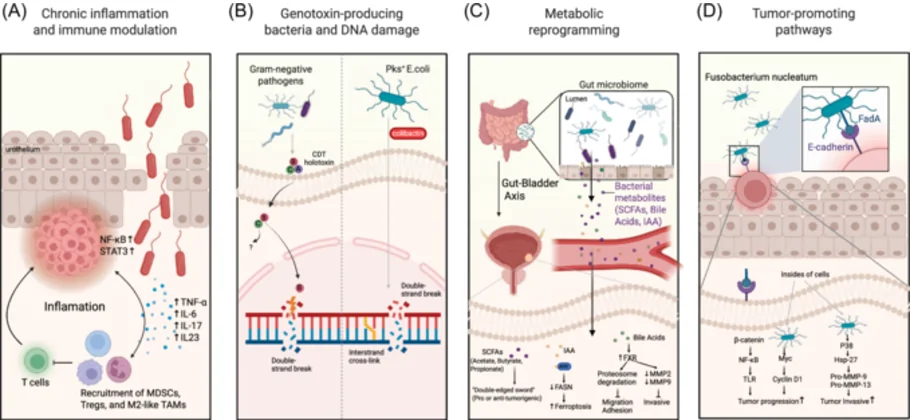
Putative mechanisms by which the microbiome promotes bladder carcinogenesis. (A) Chronic inflammation and immune modulation: dysbiotic bacteria colonize the urothelium, triggering the release of pro‐inflammatory cytokines (IL‐6, TNF‐α) via NF‐κB and STAT3 pathways. This chronic inflammation recruits immunosuppressive cells (MDSCs, Tregs, M2‐TAMs), creating a tumor‐permissive microenvironment. (B) Genotoxin‐producing bacteria and DNA damage: Pathogenic strains (e.g., pks+ *E. coli*) secrete genotoxins like colibactin, which directly induce DNA double‐strand breaks and genomic instability in urothelial cells. (C) Metabolic reprogramming: Through the “Gut‐Bladder Axis,” bacterial metabolites originating from the gut microbiome (e.g., SCFAs, Bile Acids, Indole‐3‐acetic acid/IAA) enter the circulation and reach the bladder. While some metabolites are protective, dysregulation can alter host metabolism; for instance, IAA can decrease FASN expression and promote ferroptosis, while bile acids may inhibit migration and invasion by downregulating MMP2 and MMP9 via the FXR receptor. (D) Tumor‐promoting pathways: Specific pathogens, such as *Fusobacterium nucleatum*, can adhere to urothelial cells by binding its FadA adhesin to E‐cadherin. This interaction can activate intracellular signaling pathways, such as β‐catenin, NF‐κB, and TLR. And it can upregulate the expression of oncogenes (e.g., Myc, P38, Cyclin D1) that drive tumor progression and invasion. Abbreviations: IL‐6, interleukin‐6; TNF‐α, tumor necrosis factor‐alpha; NF‐κB, nuclear factor kappa‐light‐chain‐enhancer of activated B cells; STAT3, signal transducer and activator of transcription 3; MDSCs, myeloid‐derived suppressor cells; Tregs, regulatory T cells; M2‐TAMs, M2‐polarized tumor‐associated macrophages; *E. coli*, *Escherichia coli*; SCFAs, short‐chain fatty acids; IAA, indole‐3‐acetic acid; FASN, fatty acid synthase; MMP, matrix metalloproteinase; FXR, farnesoid X receptor; TLR, Toll‐like receptor; Myc, myelocytomatosis oncogene.

The tumor‐promoting effects of this milieu are largely orchestrated by persistent pro‐inflammatory cytokine networks. Elevated levels of cytokines such as tumor necrosis factor‐alpha (TNF‐α), interleukin‐6 (IL‐6), and interleukin‐17 (IL‐17) in BCa patients promote oncogenesis by activating key signaling cascades, including nuclear factor kappa‐light‐chain‐enhancer of activated B cells (NF‐κB) and Janus kinase‐signal transducer and activator of transcription 3 (JAK‐STAT3) [98, 99, 100, 101]. Specifically, dysbiosis driven by uropathogens like *E. coli* may enhance TNF‐α secretion, which is critically involved in cancer development [102]. Elevated TNF‐α levels are observed in both serum and tumor tissues of BCa patients and correlate with advanced disease stages [103]. IL‐6 is another key mediator, associated with advanced clinical stage and poor survival [104]. It fosters an immunosuppressive tumor microenvironment (TME) by expanding myeloid‐derived suppressor cell (MDSC) populations and activating the mitogen‐activated protein kinase (MAPK) pathway to enhance their suppression of cytotoxic T‐cells [105]. The IL‐23/IL‐17 axis also plays a critical role, with high expression of IL‐17 and its receptor associated with poor prognosis [106, 107]. IL‐17 activates Stat3 via an IL‐6‐dependent mechanism, upregulating pro‐survival and pro‐angiogenic genes [108]. Targeting this axis may mitigate cancer risk by dampening Th17‐mediated inflammation [109], a process potentially influenced by microbiome‐derived metabolites as suggested by studies on gut microbiota [110].

The host's genetic constitution serves as a pivotal modifying factor, amplifying the oncogenic potential of this microbe‐induced inflammation. Polymorphisms in genes encoding major inflammatory mediators‐including TNF‐α, IL‐6, and their receptors—can alter the magnitude and persistence of the inflammatory response following infection. For instance, individuals carrying the C/C variant at the −174 locus of the IL‐6 promoter show a significantly increased risk of BCa (odds ratio [OR] = 1.77) [111]. Similarly, specific peroxisome proliferator‐activated receptor gamma (PPARG) variants and TNF‐α alleles are associated with divergent recurrence risks and aggressive phenotypes, suggesting that high‐risk allele carriers are predisposed to protracted inflammatory responses [111]. Beyond cytokine regulation, polymorphisms in pathogen recognition genes, such as the +3725G/C polymorphism in Toll‐like receptor 4 (TLR4), compromise immune surveillance against Gram‐negative bacteria, facilitating persistent colonization [112]. Concurrently, variants in immune checkpoints (e.g., cytotoxic T‐lymphocyte‐associated antigen 4 [CTLA‐4] rs231775, PD‐1 rs7421861) augment T‐cell inhibitory signals [113]. These genetic factors create a vulnerable “host soil,” wherein the introduction of “carcinogenic microbial seeds” leads to a substantially greater potential for malignant progression.

Direct physical interactions between the microbiota and the urothelium further exacerbate this inflammatory loop. Bacterial biofilms formed by uropathogens such as *E. coli* and *Enterococcus faecalis* adhere to the bladder lumen, disrupting the protective glycosaminoglycan layer [114, 115]. This adhesion causes persistent irritation and activation of epithelial cells, fueling a cycle of chronic genitourinary inflammation. The direct presence of these dense microbial communities within bladder tumor tissues has been confirmed via fluorescence in situ hybridization (FISH), providing a physical link between biofilm formation and the cancerous landscape [116].

Ultimately, this dysregulated inflammatory state drives the recruitment and modulation of immune cells, transforming the bladder into a profoundly immunosuppressive TME. Initially, the inflammatory response recruits diverse cells, including neutrophils, macrophages, and lymphocytes, which release matrix metalloproteinases (MMPs) and chemokines that support tumorigenesis [117]. Systemically, this burden is reflected in markers like an elevated neutrophil‐to‐lymphocyte ratio (NLR), a robust predictor of disease progression [118, 119]. Over time, however, chronic inflammation polarizes these infiltrates toward immunosuppressive phenotypes. Tumor‐associated macrophages (TAMs), particularly those polarized to an M2 phenotype, promote metastasis by secreting pro‐angiogenic and immunosuppressive cytokines [120]. MDSCs are also significantly enriched in BCa tissue [121, 122], expressing high levels of arginase 1 (Arg1), inducible nitric oxide synthase (iNOS), and programmed death‐ligand 1 (PD‐L1) to potently suppress T‐cell proliferation [123]. In addition to TAMs and MDSCs, regulatory T cells (Tregs) expand under chronic stimulation, inhibiting effector T‐cell function via IL‐10, TGF‐β, and CTLA‐4 [124, 125]. This environment drives T‐cell exhaustion, characterized by the upregulation of inhibitory receptors (e.g., PD‐1, T‐cell immunoglobulin and mucin‐domain containing‐3 [TIM‐3], lymphocyte‐activation gene 3 [LAG‐3]) [126]. Specific microbial interactions accelerate this exhaustion; for example, the genus *Leptotrichia* is associated with increased PD‐L1 expression on bladder tumor cells, facilitating immune escape [127]. Thus, the microbiota actively shapes the immunosuppressive landscape of the TME.

Collectively, bladder cancer‐associated dysbiosis may act as a potential driver of this immunosuppressive network. Dysbiosis‐driven shifts in the bladder and intratumoral microbiota are thought to reshape the immune landscape. Specifically, Gram‐negative taxa enriched in bladder cancer, including *F. nucleatum* [128], *Leptotrichia* [127], and genotoxin‐producing *Enterobacteriaceae* (e.g., pks+ *E. coli*) [129], promote M2 polarization of TAMs and expansion of MDSCs. This occurs through lipopolysaccharide (LPS)/TLR4 signaling and chronic inflammation triggered by bacterial genotoxins. Notably, *Leptotrichia* abundance is directly associated with increased PD‐L1 expression on tumor cells, enhancing checkpoint‐mediated T‐cell exhaustion [127]. Conversely, beneficial commensals such as *Lactobacillus* species (particularly *L. crispatus*, *L. gasseri*, and *L. johnsonii*) [85, 130, 131] and *Bifidobacterium* (specifically *B. breve* and *B. longum*) [84]—which are frequently depleted in non‐responders but enriched in Bacillus Calmette‐Guérin (BCG) responders‐generate indole derivatives. These metabolites activate the aryl hydrocarbon receptor (AhR), thereby enhancing CD8⁺ T‐cell effector function and limiting Treg differentiation [132]. Therefore, the loss of these protective taxa alongside the overgrowth of pathogens establishes a self‐reinforcing immunosuppressive circuit, suggesting that restoring microbial homeostasis represents a promising strategy to reinstate effective anti‐tumor immunosurveillance.

However, it should be noted that while the correlation between dysbiosis and the inflammatory milieu in BCa is well‐documented, the specific causal pathways driving this immunosuppressive network are largely inferred from general mucosal immunology or other cancer models, and direct mechanistic validation in the human bladder microenvironment remains to be fully established.

### 3.3 Genotoxicity and DNA damage: The role of toxin‐producing pathogens

Beyond chronic inflammation, the production of bacterial genotoxins by uropathogens represents a more direct and insidious mechanism by which the microbiota contributes to bladder carcinogenesis. These genotoxins directly compromise host DNA integrity, thereby driving genomic instability and initiating a mutagenic cascade that underlies cancer development. Among them, colibactin is a particularly potent agent. Synthesized by Enterobacteriaceae harboring the pks genomic island—such as certain strains of *E. coli* and *Klebsiella pneumoniae*—colibactin functions primarily as a DNA interstrand cross‐linking agent [129] (Figure 2B). This cross‐linking leads to the formation of interstrand cross‐links and subsequent double‐strand breaks in host epithelial cells. Although cells mount a DNA damage response to repair these lesions, persistent or erroneous repair often results in chromosomal instability, large‐scale mutations, and cell cycle arrest [129].

Another pivotal bacterial genotoxin is the cytolethal distending toxin (CDT), produced by a variety of Gram‐negative pathogens such as *E. coli*, *Campylobacter jejuni*, and *Haemophilus ducreyi* [133]. CDT is a tripartite holotoxin composed of CdtA, CdtB, and CdtC subunits. The toxin relies on the CdtA and CdtC subunits for host cell binding, which facilitates the delivery of the catalytic CdtB subunit into the nucleus. Following cellular uptake, the CdtB subunit translocates to the nucleus, where its intrinsic DNase I‐like activity introduces DNA lesions, ranging from single‐strand breaks to double‐strand breaks [133]. This genotoxic insult promptly triggers a robust DNA damage response (DDR), predominantly mediated by the ataxia‐telangiectasia mutated (ATM) kinase signaling cascade. The activation of this pathway leads to cell cycle arrest, primarily at the G2/M phase, to facilitate DNA repair [133]. Although the typical outcomes of CDT intoxication are cell death (apoptosis) or the induction of senescence, a subpopulation of damaged cells can evade these fates. The survival of these cells, which is often associated with the impairment of critical tumor suppressor pathways such as p53, enables the propagation of genetically unstable clones. This process facilitates the accumulation of pro‐oncogenic mutations, thereby contributing to the initial stages of carcinogenesis.

The cumulative effect of these genotoxic insults is a profoundly increased mutational burden in bladder epithelial cells. A vicious cycle of DNA damage, imperfect repair, and clonal expansion—further fueled by the surrounding inflammatory milieu—dramatically elevates the risk of oncogenic transformations in key genes governing cell proliferation and survival. This pathway theoretically links genotoxin‐producing bacteria in the urobiome to the initiation of BCa, positioning them as putative microbial drivers of oncogenesis.

However, a critical distinction must be made regarding the specificity of these findings. While the genotoxic potential of bacteria such as pks+ *E. coli* and the mechanisms of CDT‐induced damage are well‐established in colorectal and other gastrointestinal cancer models, direct in vivo evidence demonstrating their capacity to initiate mutagenesis specifically within human urothelial cells remains limited. Consequently, these pathways currently serve as plausible hypotheses extrapolated from other oncological fields, and dedicated bladder‐specific investigations are essential to confirm their causal role in BCa initiation.

### 3.4 Metabolic reprogramming: The gut–bladder axis

In addition to the local urobiome, the gut microbiota acts as a remote metabolic factory that may influence BCa progression by generating a variety of metabolites. This cross‐organ metabolic interaction, often termed the “gut‐bladder axis,” provides a framework for understanding microbe‐mediated cancer development (Figure 2C). However, it is imperative to distinguish between mechanisms directly validated in BCa and those inferred from other malignancies. While the latter provide a sophisticated conceptual framework, their specific role in the bladder microenvironment—characterized by unique pH, urea concentration, and urothelial barriers—remains to be rigorously proven.

Short‐chain fatty acids (SCFAs), such as butyrate, propionate, and acetate, are produced by bacterial fermentation of dietary fiber in the gut. They exhibit anti‐inflammatory and anti‐tumor properties, but can also drive the invasion of tumor cells [134]. The systemic and local metabolic impacts of SCFAs are multifaceted, and their net effects may vary depending on the metabolic state of tumor cells and the specific microenvironmental niche [134]. The evidence for SCFA‐mediated effects in BCa remains largely preliminary compared to other genitourinary cancers. For example, studies in prostate cancer have shown that gut microbiota‐derived SCFAs (acetate and butyrate) can promote tumor progression by inducing cancer cell autophagy and M2 macrophage polarization [135]. This finding provides a cautionary precedent for the genitourinary system, highlighting a potential pro‐tumor effect of SCFAs that warrants dedicated investigation in the context of BCa. In contrast, direct (though primarily in vitro) evidence suggests that butyrate‐producing probiotics like *Butyricicoccus pullicaecorum* can upregulate the expression of SCFA receptors GPR43 and GPR109B on bladder uroepithelial cells. Activation of these G‐protein‐coupled receptors can mediate anti‐inflammatory and anti‐proliferative effects, suggesting a potential protective role in bladder [136]. This context‐dependent duality extends to cancer immunotherapy. The microbiota‐derived SCFA acetate can enhance immune checkpoint inhibitor (ICI) efficacy by suppressing IL‐17A production in group 3 innate lymphoid cells (ILC3s) in hepatocellular carcinoma [137]. Conversely, the immunomodulatory impact of SCFAs is highlighted by findings in melanoma, where high systemic levels of butyrate and propionate are associated with resistance to CTLA‐4 blockade by promoting Treg activity [138]. However, the specific immunomodulatory impact of systemic SCFAs on the bladder‐specific immune microenvironment has yet to be clinically validated.

Tryptophan metabolism by gut bacteria plays a critical role in shaping immune responses and cancer outcomes. Tryptophan is converted by gut bacteria into various metabolites with divergent and context‐dependent effects. For instance, indole‐3‐aldehyde (IAld) has been shown to activate CD8⁺ T cells, promoting their proliferation and interferon‐gamma (IFN‐γ) release, thereby enhancing antitumor immunity in melanoma models [132]. The functional pleiotropy of tryptophan metabolites is further emphasized by indole‐3‐acetic acid (IAA). In a pancreatic tumor model, IAA was reported to exert an immunosuppressive effect [139]. However, in the context of BCa, recent preclinical research has identified the same metabolite, IAA, when derived from the gut bacterium *Parabacteroides distasonis*, as exhibiting a mechanistically distinct, potent tumor‐suppressive function. Li et al. demonstrated that IAA binds to and activates the AhR in BCa cells, leading to the transcriptional downregulation of fatty acid synthase (FASN). This downregulation promotes the cancer cells' sensitivity to ferroptosis [140]. This mechanism unveils a novel pathway‐the AhR‐FASN axis‐through which a gut microbiota‐derived metabolite regulates tumor lipid metabolism and induces ferroptosis, presenting a new metabolic target for intervention in BCa. Other tryptophan metabolites, such as indole‐3‐lactic acid (ILA) and indole‐3‐propionic acid (IPA), have also been shown to enhance ICI responses by modulating dendritic cell (DC) function and T cell stemness [141, 142]. In a more consistently immunosuppressive pathway, the host enzyme indoleamine 2,3‐dioxygenase 1 (IDO1), frequently upregulated in tumors, catalyzes the conversion of tryptophan to kynurenine [143], a potent immunosuppressor that inhibits T cell function and promotes immune tolerance and angiogenesis within the TME [144]. The significantly reduced plasma tryptophan levels in BCa patients underscore the systemic nature of this dysregulation [145].

Bile acids may also influence BCa progression. Elevated levels of bile acids, including chenodeoxycholic acid (CDCA), glycoursodeoxycholic acid (GUDCA), and glycochenodeoxycholic acid (GCDCA), have been detected in urine samples from BCa patients compared with healthy controls [146]. Furthermore, mechanistic studies in gastrointestinal cancers have implicated secondary bile acids, such as deoxycholic acid (DCA), in promoting DNA damage, oxidative stress, and proliferation [147], suggesting a plausible, though unconfirmed, parallel mechanism in BCa. And the farnesoid X receptor (FXR), a nuclear receptor activated by bile acid binding, has been reported to inhibit migration, invasion, and angiogenesis in BCa models through various mechanisms [148, 149]. In addition, secondary bile acids and specific bile acid species like ursodeoxycholic acid (UDCA) are enriched in ICI responders, potentially modulating CD8 + T cell activity [150]. This suggests a potential avenue for exploring bile acid metabolism in BCa immunotherapy. Another gut microbiota‐derived metabolite of interest is trimethylamine N‐oxide (TMAO), which is generated from dietary choline and l‐carnitine through a two‐step process involving gut microbial conversion to trimethylamine (TMA) followed by hepatic oxidation. It has been shown to activate CD8 + T cells and promote antitumor immunity in breast and pancreatic cancer models [151, 152]. However, elevated TMAO levels have also been associated with chronic inflammation and cancer risk in other contexts, suggesting a dual role in tumorigenesis [153].

In summary, while direct profiling of the urinary metabolome in BCa is still emerging, the established role of the gut–bladder axis provides a compelling paradigm. The well‐documented capacity of gut microbiota‐derived metabolites—such as SCFAs, tryptophan derivatives, bile acids, and TMAO—to systemically modulate host metabolism and immunity, as demonstrated in various cancer models, offers a critical framework for understanding how microbial communities might influence local carcinogenesis. However, it is crucial to emphasize that while this is an attractive hypothesis, the direct clinical evidence for these specific gut‐derived metabolites acting as primary drivers in human bladder cancer is still limited and largely extrapolated from preclinical models or other cancer types. Consequently, future research specifically dedicated to characterizing the urinary metabolome and its functional impact is not only warranted but will be essential to validate these potential pathways and uncover novel, bladder‐specific therapeutic targets.

### 3.5 Direct activation of tumor‐promoting pathways

Beyond metabolic reprogramming, specific bacteria that colonize the bladder can act directly as “triggers” for carcinogenic signals, thereby accelerating tumor progression (Figure 2D). For instance, *Fusobacterium nucleatum* has been identified in a significant proportion of BCa samples [128]. Mechanistic insights, largely derived from colorectal cancer studies, indicate that it employs surface proteins such as FadA to bind to host cells, leading to the activation of the β‐catenin signaling pathway. This activation is capable of driving cellular proliferation and stimulates NF‐κB, resulting in a chronic inflammatory response and the release of cytokines like IL‐6 and IL‐8 [154]. This non‐metabolite‐mediated mechanism nonetheless reshapes the local TME and affects host cell metabolism. Notably, the immunomodulatory potential of *F. nucleatum* is not limited to one cancer type; it has been shown to sensitize microsatellite‐stable colorectal cancer to immunotherapy by modulating PD‐L1 expression [155]. This highlights a broader mechanistic principle whereby specific bacteria can influence the TME and response to treatment, the relevance of which to BCa warrants further study. Another example is *Eubacterium* spp., which, when co‐cultured with BCa organoids, promotes tumor cell growth via the ECM1/MMP9 pathway, a cascade critically involved in cell proliferation, invasion, and metastasis [82]. Furthermore, pathogenic strains of *E. coli* producing Cytotoxic Necrotizing Factor 1 (CNF1) constitutively activate Rho GTPases. CNF1 activates NF‐κB through the Akt/IKK signaling pathway, thereby enhancing cell survival by upregulating anti‐apoptotic proteins such as Bcl‐2 and Bcl‐XL. Additionally, CNF1 induces the production of pro‐inflammatory cytokines (e.g., IL‐6, IL‐8, TNF‐α) and cyclooxygenase‐2 (COX‐2). These effects may collectively promote cell survival, inflammatory responses, and tumor progression within the tumor [156] (Table 2).

**TABLE 2:** The possible mechanisms by which the microbiome influences bladder carcinogenesis.

Mechanism category	Key microbe/metabolite example	Effector molecule/toxin	Downstream pathway & oncogenic effect	Representative references
**Chronic Inflammation and Immune Modulation**	Dysbiosis(increased opportunistic pathogens)	TNF‐α, IL‐6, IL‐17, ROS/RNS, IL‐10, TGF‐β	Activation of signaling pathways: NF‐κB, STAT3 Recruitment/Polarization of immunosuppressive cells: MDSCs, TAMs (M2 phenotype), Tregs Inhibition of T‐cell function: Induction of T‐cell exhaustion Genetic damage: Induction of DNA damage, contributing to gene mutations	[91, 97, 98, 99, 100, 101, 105, 108, 117, 120, 121, 122, 123, 124, 125, 126]
	*Schistosoma haematobium*	Carcinogenic N‐nitrosamines	Perpetuation of a pro‐tumorigenic inflammatory environment	[93, 94]
	*Leptotrichia* (urine)	PD‐L1	Increased PD‐L1 expression on bladder tumor cells, facilitating immune evasion	[127]
**Genotoxins and DNA Damage**	pks+ *E. coli*, *Klebsiella pneumoniae*	Colibactin	Functions as a DNA interstrand cross‐linking agent, causing double‐strand breaks and genomic instability	[129]
	*E. coli*, *Campylobacter jejuni, Haemophilus ducreyi*	Cytolethal Distending Toxin (CDT)	The CdtB subunit acts as a DNase, causing DNA lesions and activating the ATM‐mediated DNA damage response, leading to cell cycle arrest and propagation of mutated cells	[133]
**Metabolic Reprogramming**	*Parabacteroides distasonis* (gut)	Indole‐3‐acetic acid (IAA)	IAA activates AhR, downregulating FASN and sensitizing BCa cells to ferroptosis.	[140]
	Tryptophan metabolites	Indole‐3‐aldehyde (IAld), Indole‐3‐lactic acid (ILA), Indole‐3‐propionic acid (IPA)	IAld activates CD8⁺ T cells, enhancing anti‐tumor immunity. ILA and IPA enhance ICI responses by modulating DC function and T cell stemness	[132, 141, 142]
	Trimethylamine N‐oxide (TMAO)	‐	Activates CD8⁺ T cells and enhances anti‐tumor immunity in breast and pancreatic cancer. Associated with chronic inflammation and cancer risk.	[151, 152, 153]
	Bile Acids	Deoxycholic acid (DCA)	DCA may promote DNA damage and oxidative stress; Bile Acids can activate FXR to inhibit migration, invasion, and angiogenesis in BCa.	[147, 148, 149]
	Short‐chain fatty acids (SCFAs)	butyrate, propionate, acetate	Acetate and butyrate can promote prostate cancer progression by inducing cancer cell autophagy and M2 macrophage polarization via TLR3‐triggered activation of NF‐kB and MAPK signalings Butyrate‐producing probiotics like *Butyricicoccus pullicaecorum* can upregulate the expression of SCFA receptors GPR43 and GPR109B on bladder uroepithelial cells. Activation of these GPRs can mediate anti‐inflammatory and anti‐proliferative effects. The microbiota‐derived SCFA acetate can enhance ICI efficacy by suppressing IL‐17A production in group 3 innate lymphoid cells (ILC3s) in hepatocellular carcinoma High systemic levels of butyrate and propionate are associated with resistance to CTLA‐4 blockade by promoting regulatory T cell (Treg) activity and limiting dendritic cell maturation in melanoma	[134, 135, 136, 137, 138]
**Tumor‐Promoting Pathways**	*Fusobacterium nucleatum*	FadA adhesin	Binds to host cells, activating β‐catenin signaling to drive proliferation and activating NF‐κB to induce pro‐inflammatory cytokines (IL‐6, IL‐8).	[154]
	*Eubacterium* spp.	‐	Promotes tumor cell growth via the ECM1/ERK1/2/MMP9 pathway, enhancing proliferation, invasion, and metastasis.	[82]
	Pathogenic *E. coli*	Cytotoxic Necrotizing Factor 1 (CNF1)	Constitutively activates Rho GTPases, stimulating the Akt/IKK/NF‐κB pathway to enhance cell survival, anti‐apoptotic protein expression, and pro‐inflammatory signaling.	[156]

Abbreviations: ATM, ataxia‐telangiectasia mutated; AhR, aryl hydrocarbon receptor; BCa, bladder cancer; CNF1, cytotoxic necrotizing factor 1; COX‐2, cyclooxygenase‐2; CDT, cytolethal distending toxin; CTLA‐4, cytotoxic T‐lymphocyte‐associated antigen 4; DCA, deoxycholic acid; DC, dendritic cell; ECM1, extracellular matrix protein 1; ERK, extracellular signal‐regulated kinase; *E. coli*, *Escherichia coli*; FASN, fatty acid synthase; FXR, farnesoid X receptor; GPR, G‐protein‐coupled receptor; IAA, indole‐3‐acetic acid; IAld, indole‐3‐aldehyde; ILA, indole‐3‐lactic acid; IPA, indole‐3‐propionic acid; ICI, immune checkpoint inhibitor; IL, interleukin; ILC3s, group 3 innate lymphoid cells; MDSCs, myeloid‐derived suppressor cells; MAPK, mitogen‐activated protein kinase; MMP, matrix metalloproteinase; NF‐κB, nuclear factor kappa‐light‐chain‐enhancer of activated B cells; PD‐L1, programmed death‐ligand 1; ROS, reactive oxygen species; RNS, reactive nitrogen species; SCFAs, short‐chain fatty acids; STAT3, signal transducer and activator of transcription 3; TNF‐α, tumor necrosis factor‐alpha; TGF‐β, transforming growth factor‐beta; TAMs, tumor‐associated macrophages; Tregs, regulatory T cells; TMAO, trimethylamine N‐oxide; TLR, Toll‐like receptor.

In summary, with the exception of *Eubacterium* spp., whose mechanism has been functionally validated in BCa organoids, many of these direct signaling interactions, particularly those involving *F. nucleatum*, are currently extrapolated from colorectal cancer studies; thus, their precise role in modulating urothelial signaling pathways requires distinct confirmation.

## 4 CLINICAL TRANSLATION: FROM BIOMARKERS TO THERAPEUTICS

### 4.1 Diagnostic and prognostic applications: Utilizing microbial signatures for risk stratification

The identification of distinct microbial ecosystems in BCa patients establishes the microbiome as a promising reservoir for novel biomarkers. However, to effectively harness this potential, it is critical to distinguish between biomarkers derived from tissue versus those detected in urine. Tissue‐derived microbial signatures provide direct insights into the tumor microenvironment, often revealing bacteria that intimately interact with urothelial cells to drive carcinogenesis. Yet, the invasive nature of tissue biopsy limits its utility for routine screening. In contrast, urinary biomarkers offer a superior translational advantage as a “liquid biopsy,” enabling repeated, non‐invasive sampling for diagnosis and longitudinal surveillance. Despite this promise, a major challenge in translating these findings lies in the heterogeneity introduced by sampling methods and host factors. Variations between midstream voided urine and catheterized samples can introduce significant variability in microbial composition due to the inclusion of distal urethral or genital flora. Furthermore, host‐specific variables such as sex, age, and smoking status act as confounding factors that may mask disease‐specific signals. Therefore, establishing standardized collection protocols and rigorous adjustment for host factors are prerequisites for clinical implementation.

Amidst this heterogeneity, certain microbial signals have demonstrated robustness across studies, bridging the gap between correlation and diagnostic power. While broad dysbiosis, characterized by the enrichment of pathogens like *Enterobacter* and *Cupriavidus* alongside the depletion of commensals such as *Lactobacillus*, is frequently reported in urine, specific taxa show higher consistency. This translational potential is best exemplified by *Eubacterium* sp. CAG:581, a bacterium mechanistically linked to tumor progression via the ECM1/MMP9 pathway [82]. In a large validation cohort of 406 patients, this specific signature overcame background variability to achieve an AUC of 0.79 for predicting 3‐year occurrence [82], marking a pivotal step from a candidate marker to a validated tool for early diagnosis.

Beyond initial diagnosis, microbial profiles serve as critical indicators for prognosis and recurrence risk, although findings regarding community diversity require nuanced interpretation. Current evidence regarding alpha diversity and clinical outcomes is conflicting. A recent large‐scale study comprising 170 BCa patients (125 primary and 45 recurrent cases) provided further insights into microbial distinctions. Following propensity score matching, analysis of 39 matched pairs demonstrated significantly higher alpha diversity in primary BCa cases than in recurrent cases, as indicated by Chao1 indices [80]. Beta diversity analysis also revealed distinct microbial community structures between the two groups (ADONIS, *p* = 0.004). Other investigations have observed the opposite, reporting that high urinary microbial diversity post‐treatment correlates with shorter recurrence‐free survival [71, 79]. These discrepancies likely reflect the aforementioned heterogeneity in sampling methods, clinical status, and patient cohorts, suggesting that relying solely on broad diversity metrics is insufficient for risk stratification.

While diversity metrics are inconsistent, specific microbial signatures present at the time of initial surgery appear more consistently associated with future recurrence. Prospective analyses of urine samples collected during the initial transurethral resection of bladder tumor (TURBT) indicate that patients who later experience recurrence often exhibit an enrichment of genera such as *Corynebacterium*, *Anaerococcus*, *Muribaculaceae*, and *Cutibacterium*. In contrast, non‐recurrent groups are characterized by species or genera such as *Ruminococcus torques*, *Brevundimonas*, and *Pseudomonas* [83]. The temporal stability of these signatures is notable, *Corynebacterium* remained more abundant in urine samples collected at the 1‐year cystoscopy from patients with recurrent disease [83], suggesting its potential involvement in tumor recurrence (Table 1).

To translate these diverse findings into real‐world clinical practice, future development must transition from the broad 16S rRNA sequencing used in discovery phases to rapid, cost‐effective detection methods. The identification of high‐consistency markers, such as *Eubacterium* sp. CAG:581 and *Lactobacillus*, opens avenues for developing targeted assays, such as polymerase chain reaction (PCR) panels or enzyme‐linked immunosorbent assay (ELISA)‐based tests, which could be integrated into routine urinalysis. Furthermore, developing standardized “dysbiosis indices” that quantify the ratio of pathogenic to protective genera may offer higher diagnostic sensitivity than single‐pathogen detection. Such microbiome‐based tools could complement existing surveillance strategies like cystoscopy, offering a non‐invasive means to refine risk assessment and guide personalized interventions during follow‐up.

### 4.2 The microbiome as a predictor of therapeutic response

The host microbiome has emerged as a critical determinant of therapeutic efficacy in BCa. Beyond simple correlation, specific microbial profiles in both the bladder and the gut serve as robust predictors of response to immunotherapy and chemotherapy, offering a non‐invasive avenue for patient stratification and personalized treatment planning.

#### 4.2.1 Microbiome predictors of BCG immunotherapy efficacy

BCG, a live attenuated strain of *Mycobacterium bovis*, represents the standard adjuvant immunotherapy for intermediate‐risk NMIBC and high‐risk NMIBC [9, 10]. Despite its established efficacy, treatment failure remains a significant clinical challenge, with 40% of patients experiencing disease recurrence and approximately 20% progressing to MIBC [157]. Emerging evidence strongly implicates the local bladder dysbiosis (encompassing both fluid‐based and tissue‐resident microbiota) as a potential modulator of BCG therapeutic efficacy (Figure 3A).

**FIGURE 3:**
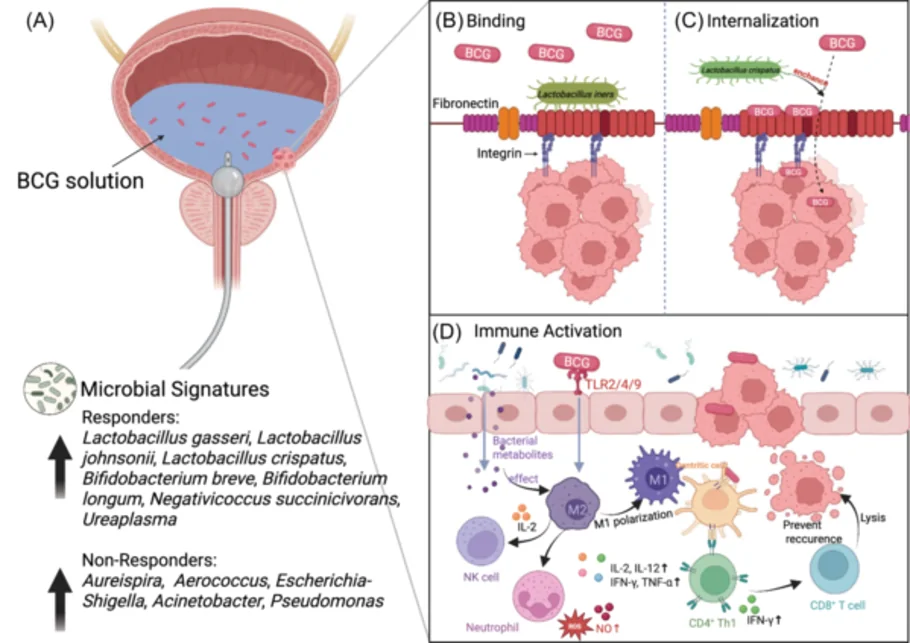
Proposed mechanisms modulating BCG immunotherapy efficacy via the urinary microbiome. The efficacy of BCG intravesical instillation relies on a multistep cascade involving adhesion, internalization, and immune activation, each of which can be influenced by specific microbial signatures. (A) Overview and microbial signatures. Intravesical BCG solution is administered into the bladder. Clinical responders are characterized by an enrichment of beneficial taxa (e.g., *Lactobacillus gasseri*, *Lactobacillus crispatus*), whereas non‐responders show an enrichment of potential pathogens (e.g., *Escherichia‐Shigella*, *Pseudomonas*). (B) Competitive inhibition of adhesion. BCG attachment to urothelial fibronectin via its FAP is a prerequisite for therapeutic action. Commensal bacteria, such as *Lactobacillus iners*, may bind to fibronectin and spatially compete with BCG for adhesion sites, potentially reducing the initial therapeutic load attached to the bladder wall. (C) Modulation of internalization. Following adhesion, BCG must be internalized by urothelial carcinoma cells to effectively trigger antigen processing. Specific beneficial strains, such as *Lactobacillus crispatus*, have been postulated to enhance BCG internalization, thereby amplifying the intracellular signals required for immune recognition. (D) Orchestration of immune activation. BCG triggers robust innate immunity via TLR2/4/9, inducing M1 macrophage polarization, neutrophil recruitment (ROS/NO production), and NK cell activation. This creates a cytokine milieu (IL‐2, IL‐12, IFN‐γ, TNF‐α) that facilitates dendritic cell maturation and the subsequent activation of adaptive CD4 + Th1 and CD8+ cytotoxic T cells for tumor lysis. “Responders” are characterized by an enrichment of beneficial taxa that may synergize with these pathways, whereas “Non‐responders” are enriched with potential pathogens that may fail to support or actively suppress this anti‐tumor immune loop. BCG, Bacillus Calmette‐Guérin; FAP, fibronectin attachment protein; TLR, Toll‐like receptor; ROS, reactive oxygen species; NO, nitric oxide; NK cell, natural killer cell; IL, interleukin; IFN‐γ, interferon‐gamma; TNF‐α, tumor necrosis factor‐alpha; Th1, Type 1 helper T cell.

Recent clinical studies have begun to delineate distinct microbial signatures associated with BCG response. A pivotal study by Knorr et al. demonstrated that the urothelial microbiome exhibits compositional differences between responders and non‐responders. Using both 16S rRNA and shotgun metagenomic sequencing on tissue samples, they found that microbial species richness and community structure were significantly different between the two groups. Notably, *L. gasseri* and *L. johnsonii* were significantly enriched in the BCG responders [131]. This was functionally validated in vitro, where co‐culture with *L. crispatus* enhanced the internalization of BCG by urothelial cancer cells, suggesting a potential probiotic mechanism to augment BCG efficacy [131]. This finding is supported by independent studies, which also identified *Lactobacillus* spp., particularly *L. crispatus*, as enriched in the bladder tissue samples of responders and demonstrated that its co‐culture significantly increases BCG internalization into urothelial carcinoma cells [85, 130]. Furthermore, shotgun metagenomics on tissue samples identified metabolic pathways such as assimilatory sulfate reduction and the reductive citrate cycle as being enriched in responders, hinting at a metabolic interplay between the resident microbiota and BCG's mechanism of action [131].

The association of other bacterial genera with BCG response appears complex and may vary by sample type. Analysis of tumor tissue revealed conflicting associations for *Corynebacterium*, one study reports an enrichment of *Corynebacterium* and *Pseudomonas* in responders [86], others have found *Corynebacterium* enriched in non‐responders [85]. This dichotomy may be mechanistically explained by “molecular mimicry.” Since Corynebacterium belongs to the same phylum (*Actinobacteria*) as the BCG strain of *M. bovis* and shares cell wall mycolic acids, its presence may prime the local immune system via cross‐reactivity [158], thereby enhancing the anti‐tumor response. Conversely, in different cohorts, its abundance might simply reflect a general dysbiotic burden.

Furthermore, BCG therapy itself dynamically remodels the tumor microbiome. Analysis of paired tumor samples revealed that treatment alters the microbial landscape, with *M. bovis* found to be paradoxically enriched in tumors after BCG failure [86]. This suggests that the mere physical persistence of the bacillus is insufficient for therapeutic success; rather, chronic persistence without clearance may be associated with a state of immune tolerance rather than effective anti‐tumor immunity.

Concurrently, longitudinal studies tracking the urinary bladder microbiome during BCG therapy provide insights into dynamic host‐microbe interactions. Boban et al. serially collected catheterized urine samples throughout the six induction cycles of BCG. They observed a significant decrease in microbial biodiversity (Shannon Index) following the first BCG instillation, suggesting a disruptive effect of BCG on the bladder microbial community [159]. Another study of NMIBC patients treated with intravesical therapy (IVT), including BCG (*n* = 27) or gemcitabine (*n* = 2), also observed a significant decrease in bacterial richness following treatment. This study further found that disease recurrence correlated strongly with a higher relative abundance of *Aerococcus* (*p* < 0.01); conversely, recurrence‐free patients showed significant enrichment of *Ureaplasma* (*p* = 0.01) and *Escherichia/Shigella* (*p* = 0.05) [81]. More importantly, Boban et al.'s study indicated that the pre‐therapy microbial composition in urine already differed between eventual responders and non‐responders. Non‐responders had a significantly higher abundance of the genus *Aureispira* in pre‐therapy urine, while responders were enriched with *Negativicoccus succinicivorans* [159]. The abundance of *Aureispira* decreased eightfold by the end of therapy in the urine of non‐responders, indicating that BCG treatment dynamically alters the microbiome in a response‐dependent manner.

These studies collectively reinforce the potential predictive value of specific taxa, while also revealing inconsistencies that necessitate further investigation. Adding another layer, Min et al. focused on the metabolic potential of the urobiome. Their analysis of urine samples revealed that BCG responders exhibited increased activity in microbial metabolic pathways related to quinolone biosynthesis post‐treatment [84]. Furthermore, specific beneficial bacteria, particularly *Bifidobacterium breve* and *Bifidobacterium longum*, were significantly associated with responders in urine and correlated with prolonged recurrence‐free survival [84]. A predictive model based on the abundance of these microbes in urine showed good accuracy in forecasting BCG response, highlighting the potential of the microbiome as a predictive biomarker [84].

Mechanistically, although the precise pathways underlying these clinical associations are not yet fully understood, current research points to three distinct biological mechanisms by which the urobiome modulates BCG efficacy: First, Modulation of Bacterial Adhesion: A critical initiating event in BCG's antitumor action involves its adhesion to the bladder wall through the fibronectin attachment protein (FAP), followed by internalization and the activation of a local immune response [160, 161]. Certain commensals may interfere with colonization; for example, *L. iners* exhibits a high affinity for fibronectin [162], suggesting that its abundance may competitively block binding sites and reduce BCG responsiveness (Figure 3B). Second, Enhancement of Internalization: specific strains such as *L. crispatus* can enhance the uptake of BCG by BCa cells [85, 130, 131], which may partly explain the beneficial association of Lactobacillus enrichment in responders (Figure 3C). Third, Immune Activation and Metabolic Modulation: The metabolic output of the urobiome directly shapes the local immune microenvironment (Figure 3D). Studies have shown that key genes involved in tryptophan metabolism (e.g., indole‐3‐pyruvate decarboxylase) are significantly upregulated in the tumor microbiome of BCG responders [131]. Indole metabolites, such as IAld produced by bacteria including *Lactobacillus* [163, 164], have been shown to activate the AhR in CD8⁺ T cells, promoting the production of IFN‐γ and granzyme B to enhance anti‐tumor immunity [132]. Beyond metabolic regulation, the urobiome may also influence therapeutic efficacy by modulating mucosal inflammation, potentially through the suppression of pathways such as NF‐κB and cytokines, including IL‐6 and IL‐8 [165].

In summary, the interplay between the urobiome and BCG is multifaceted. Although growing clinical evidence points to a compelling association between microbial composition and treatment outcomes, establishing definitive causality and elucidating the underlying molecular mechanisms require further investigation. Future research holds the promise of not only yielding biomarkers for predicting response but also informing novel strategies to enhance BCG efficacy through targeted microbiome modulation.

#### 4.2.2 Predictive value of microbiota in chemotherapy and ICIs outcomes

ICIs, including anti‐PD‐1/PD‐L1 and anti‐CTLA‐4 monoclonal antibodies, reactivate antitumor immune responses by blocking tumor immune evasion pathways. While ICIs and chemotherapy are standard treatments for advanced MIBC, response rates vary. Much of the current understanding regarding microbial modulation of therapy response stems from the gut microbiome. Parallel insights from these systemic interactions offer a framework for understanding the potentially more direct role of the urobiome.

Clinical and preclinical studies have consistently demonstrated a correlation between gut microbiome composition and ICI efficacy in BCa. An analysis of stool samples from a cohort of 42 patients with MIBC receiving neoadjuvant pembrolizumab in the PURE‐01 trial (NCT02736266) [166, 167], which included 23 responders, revealed a distinct microbial signature associated with treatment outcome. Responders showed an enrichment of the genus *Sutterella*, whereas non‐responders had a higher abundance of *Ruminococcus bromii* (*R. bromii*) [168]. The causal role of *R. bromii* was further supported by preclinical models, in which its administration directly impaired the response to anti‐PD‐1 therapy [168]. Specific gut bacteria, including *B. pseudolongum*, *L. johnsonii*, and *Olsenella* species, enhance the antitumor efficacy of CTLA‐4 and PD‐1 inhibitors in mouse models of BCa through a mechanism dependent on the metabolite inosine [169]. In addition, studies have established the role of gut microbiota in other cancers‐where higher abundances of *Akkermansia*, *Enterococcus*, *Clostridiales*, *Ruminococcaceae*, *Faecalibacterium*, and *Bifidobacterium* are associated with improved ICI outcomes [170, 171, 172]. Similarly, the gut microbiome may influence chemotherapy response. A separate study investigating the influence of the gut microbiome on the response to neoadjuvant chemotherapy involved 142 BCa patients and 48 healthy controls. The analysis identified a significant association between a poor response to chemotherapy and a higher relative abundance of gut *Bacteroides* (26.95% vs. 18.93%, *p* < 0.01) and *Pseudomonas* (2.18% vs. 1.67%, *p* = 0.03) [173]. Specifically, this effect appeared independent of short‐chain fatty acid levels, which showed no significant difference between responders and non‐responders.

Despite these systemic insights, the urinary microbiota resides in direct contact with the urothelium, offering a unique platform for local immune modulation and “crosstalk” within the tumor microenvironment. While the gut constitutes a larger reservoir, the local urobiome may exert precise, spatial influence on bladder mucosal immunity. Evidence for this local interaction is emerging. Chen et al. demonstrated that the urogenital microbiome composition significantly differs between PD‐L1–positive and PD‐L1–negative male NMIBC patients [127]. Notably, the genus *Leptotrichia*, along with *Roseomonas* and *Propionibacterium*, was significantly enriched in PD‐L1‐positive patients. This association suggests a potential mechanistic link where specific urinary taxa may drive local inflammation or T‐cell exhaustion, thereby upregulating immune checkpoints on the urothelium. Although the use of PD‐L1 expression as a standalone predictive marker remains debated [174], its correlation with specific urinary taxa underscores a functional host‐microbe interaction that could influence the “immune‐hot” or “immune‐cold” status of the bladder. Furthermore, the consistent association between antibiotic use and poorer survival in ICI‐treated urothelial carcinoma patients [175, 176, 177] likely reflects a dual perturbation of both gut and urinary ecosystems. Collectively, while direct evidence remains more limited compared to the gut, these findings highlight the urobiome as a promising, locoregional modulator of ICI response that warrants targeted investigation beyond systemic surrogates.

### 4.3 Targeting the microbiome: Probiotics, antibiotics, and novel interventions

The growing recognition of the microbiome's role in bladder carcinogenesis and therapeutic response has accelerated the development of microbiome‐modulating strategies as essential adjuncts to standard care. These interventions aim to correct dysbiosis and foster a microbial milieu conducive to treatment success.

#### 4.3.1 Microbiome‐conscious antibiotic stewardship

The administration of antibiotics, while necessary for treating infections, acts as a double‐edged sword in oncology due to its potential to disrupt the delicate equilibrium of the gut and urobiomes. Substantial clinical evidence indicates that antibiotic (ATB) use during ICI administration is consistently associated with poorer outcomes in patients with urothelial carcinoma (UC) [175, 176, 177] and other malignancies [178, 179, 180, 181]. Specifically, broad‐spectrum antibiotics administered prior to ICI initiation significantly reduce overall survival, whereas narrow‐spectrum antibiotics show no comparable detrimental effect [182, 183]. This negative association is largely linked to ATB‐induced dysbiosis, which disrupts the host immune system's ability to mount an effective anti‐tumor response.

Specific clinical trials have provided granular data reinforcing this negative association. In the PURE‐01 study, which involved 148 patients with muscle‐invasive bladder cancer (MIBC) receiving neoadjuvant pembrolizumab, concomitant ATB therapy, particularly with fluoroquinolones, was associated with significantly lower rates of pathologic complete response and reduced 24‐month recurrence‐free survival compared to non‐users [175]. Similarly, a retrospective study of 67 patients with metastatic UC treated with pembrolizumab found that those exposed to antibiotics had drastically shorter median progression‐free survival (median: 1.1 vs. 8.9 months; *p* < 0.001) and overall survival (median: 2.3 vs. 19.5 months; *p* < 0.001) [176]. A comprehensive meta‐analysis encompassing 5095 ICI‐treated UC patients confirmed these findings, demonstrating that ATB use was significantly associated with worse overall survival and progression‐free survival [177].

The mechanisms underlying this phenomenon are complex. Beyond the well‐established systemic immunosuppression resulting from antibiotic‐induced dysbiosis of the gut or urinary tract epithelium, emerging research suggests that it may also involve the direct suppression of intratumoral bacteria. Evidence indicates that intracellular bacteria within the urothelial carcinoma microenvironment can induce antibacterial immune functions and upregulate the activity of antitumor T cells via activation of the CD74‐MIF/COPA signaling pathway. Antibiotics may abolish this process by eliminating these intracellular bacteria, thereby compromising the clinical benefit of immune checkpoint inhibitors [184].

However, the impact of antibiotic exposure on treatment regimens that combine chemotherapy with immunotherapy (Chemo‐IO) or on conventional chemotherapy alone remains a subject of debate, with studies reporting conflicting outcomes. A comparative study of 950 non‐small‐cell lung cancer (NSCLC) patients demonstrated that antibiotic use was a strong predictor of worse overall survival and progression‐free survival in the pembrolizumab monotherapy cohort but had no significant impact on outcomes in the chemotherapy‐alone cohort [185]. This finding aligns with an international multicenter study of 302 patients with stage IV NSCLC treated with first‐line Chemo‐IO, which found that prior antibiotic exposure was not associated with differences in survival outcomes [186]. Similarly, other studies reported no significant association between ATB use and survival outcomes in patients receiving docetaxel [179]. Conversely, a large‐scale retrospective analysis of 2028 patients with advanced NSCLC, including 769 patients treated with Chemo‐IO, reported that antibiotic exposure was associated with a lower objective response rate (27% vs. 40%; *p* = 0.001) and shorter overall survival [187]. Similar negative associations have been observed in locally advanced head and neck cancer treated with chemoradiotherapy [188]. Given that bladder cancer is uniquely influenced by a local urobiome, in addition to the systemic gut microbiome, the specific impact of antibiotics on Chemo‐IO outcomes in BCa patients urgently requires dedicated, BCa‐specific clinical investigation.

#### 4.3.2 Probiotics and live biotherapeutic products (LBPs)

In contrast to the deleterious effects of antibiotics, the administration of specific probiotics and LBPs holds significant promise for enhancing treatment efficacy. In the context of NMIBC, *Lactobacillus* species have been identified as key modulators of BCG immunotherapy. Experimental evidence shows that *Lactobacillus crispatus* promotes BCG internalization into urothelial carcinoma cells [85, 130], a critical step for efficacy. This translational potential is supported by a key clinical trial in which oral administration of *Lactobacillus casei* for 1 year following intravesical epirubicin chemotherapy significantly improved the 3‐year recurrence‐free survival rate compared to epirubicin alone (74.6% vs. 59.9%; *p* = 0.0234) [189]. Furthermore, preclinical studies reinforce this potential: in orthotopic mouse models of BCa, direct intravesical instillation of Lactobacillus casei Shirota or *L. rhamnosus* GG (LGG) was more effective than BCG in reducing tumor incidence and progression [190, 191].

Beyond local therapy, probiotics are demonstrating the capacity to enhance systemic immunotherapy responses. *Lactobacillus rhamnosus* Probio‐M9 has been shown to synergize with anti‐PD‐1 therapy by restoring antibiotic‐disrupted gut microbiota [192]; this effect is linked to increased production of butyric acid and subsequent elevation of systemic metabolites like α‐ketoglutaric acid, which promote the infiltration of cytotoxic T lymphocytes (CTLs) into the tumor microenvironment [193]. Similarly, *Lactobacillus gallinarum* improves anti‐PD‐1 efficacy by producing indole‐3‐carboxylic acid (ICA), which inhibits the IDO1/Kyn/AhR axis, thereby suppressing regulatory T cell (Treg) differentiation and enhancing CD8 + T cell function [194]. Furthermore, LGG can induce a type I interferon response in dendritic cells via the cyclic GMP‐AMP synthase (cGAS)/stimulator of interferon genes (STING) pathway, enhancing T cell cross‐priming and anti‐tumor immunity when combined with ICIs [195]. The potential for synergy extends to other commensals, including *Bifidobacterium bifidum*, *Faecalibacterium prausnitzii*, *Blautia coccoides*, and *Alistipes onderdonkii*, all of which have been found to augment ICI responses in various cancer models [196, 197, 198, 199]. Recent findings also suggest that specific combinations, such as levofloxacin with *Clostridium butyricum* MIYAIRI 588, can enhance ICI efficacy, highlighting the potential of tailored antibiotic‐probiotic regimens [200].

Probiotics may also boost chemotherapy outcomes. Mixtures containing *Lactobacillus casei* Shirota and *Bifidobacterium breve* have been shown to activate antigen presentation and recruit cytotoxic T cells in BCa models treated with gemcitabine and cisplatin [201]. Other strains, such as *Lactobacillus acidophilus* and *Lactobacillus casei*, may sensitize tumor cells to apoptosis induced by 5‐fluorouracil [202], and the bacteriocin nisin can enhance the efficacy of doxorubicin [203].

Despite these promising findings, translating LBPs into clinical practice faces significant hurdles. Safety is a primary concern, as introducing live bacteria to immunocompromised cancer patients carries risks of infection. Manufacturing and regulatory standardization are also challenging due to batch‐to‐batch variability in bacterial viability. Biologically, the native microbiome often resists the colonization of exogenous probiotics, limiting their long‐term effectiveness. Additionally, the potential transfer of antibiotic resistance genes to pathogens remains a critical safety issue requiring rigorous screening.

#### 4.3.3 Novel precision interventions: Diet, FMT, and metabolic targeting

Moving beyond conventional supplementation, the field is advancing toward precision interventions that target the gut‐bladder axis. Dietary modulation represents a scalable, non‐invasive strategy to indirectly modify the urinary microenvironment. A prospective cohort study in patients with metastatic urothelial carcinoma receiving immune checkpoint blockade (ICB) found that high dietary fiber intake (≥17.5 g/day) was significantly associated with longer progression‐free survival (HR 0.39, 95% CI 0.16–0.93, *p* = 0.03) [204]. This observational evidence is currently being validated in randomized controlled trials (e.g., NCT04645680) designed to define the causal impact of high‐fiber diets on anti‐tumor immunity [205].

For patients with refractory dysbiosis, fecal microbiota transplantation (FMT) offers a radical approach to “reset” the ecosystem. In a phase I trial for advanced melanoma, the combination of healthy‐donor FMT with anti‐PD‐1 therapy demonstrated safety and an encouraging objective response rate of 65% [206]. Longitudinal analyses confirmed that responders successfully engrafted donor‐derived microbial strains, shifting their gut microbiome toward an immunogenic phenotype that enhanced anti‐tumor immunity [206]. While promising, FMT presents challenges in scalability and standardization compared to dietary interventions.

Emerging strategies also include the use of targeted antimicrobials. To avoid the detrimental effects of broad‐spectrum antibiotics, future approaches will employ bacteriophage therapy or narrow‐spectrum agents to selectively eliminate tumor‐promoting pathogens (e.g., genotoxin‐producing *E. coli*) while preserving beneficial commensals [175, 176, 177]. However, the clinical implementation of these precision tools is fraught with technical and regulatory complexity. For bacteriophage therapy, the rapid emergence of bacterial resistance and the host immune system's clearance of phage particles (via the reticuloendothelial system) significantly limit in vivo efficacy. Moreover, regulatory frameworks currently struggle to classify phages, which are evolving biological entities and fitting them poorly into traditional drug approval pipelines. Similarly, while FMT is effective, it lacks standardization; the “undefined” nature of fecal material poses risks of transmitting undetected pathogens or pathogenic phenotypes, underscoring the urgent need to transition towards defined microbial consortia where safety profiles are chemically and genomically fully characterized.

Finally, therapeutic exploitation of microbial metabolites remains a key frontier. Research is actively targeting the inhibition of bacterial enzymes responsible for procarcinogenic compounds like colibactin [129], while seeking to harness beneficial metabolites from tryptophan metabolism or SCFAs [131, 132]. However, translating insights from targeting these host‐microbe shared metabolic pathways into effective therapies requires a cautious, precision medicine approach. The experience with IDO1 inhibitors serves as a critical lesson: despite promising results in preclinical studies and early‐phase clinical trials (ECHO‐202/KEYNOTE‐037) that highlighted the potential of combining IDO1 inhibitors (Epacadostat) with PD‐1 inhibitors [207], the phase 3 ECHO‐301/KEYNOTE‐252 trial demonstrated that combining the IDO1 inhibitor epacadostat with pembrolizumab failed to improve progression‐free survival or overall survival compared to pembrolizumab monotherapy in patients with advanced melanoma [208]. This failure serves as a critical cautionary tale for the entire field of microbiome‐based metabolic therapies: it highlights the perils of targeting a single pathway without fully accounting for functional redundancy (e.g., compensatory activation of tryptophan 2,3‐dioxygenase [TDO2] or interleukin 4 induced 1 [IL4I1]) and the lack of patient stratification [209]. It underscores that future trials targeting microbial metabolites must rely on robust biomarkers to confirm target engagement and select the specific patient subgroups likely to benefit. Without such precision, broad metabolic modulation risks repeating the expensive failures of the past (Table 3).

**TABLE 3:** Strategies for targeting the microbiome to improve cancer treatment response.

Strategy category	Specific example	Potential mechanism	Targeted therapy	Level of evidence	Representative references
**Probiotics and Live Biotherapeutic Products (LBPs)**	*Lactobacillus casei* (oral)	Improved 3‐year recurrence‐free survival when combined with intravesical epirubicin chemotherapy	Chemotherapy (Epirubicin)	Clinical Trial	[189]
	*Lactobacillus crispatus*	Enhances BCG internalization into bladder cancer cells	BCG	Preclinical/Clinical Association	[85, 130, 131]
	*Lactobacillus rhamnosus* GG, *Lactobacillus casei* Shirota (intravesical instillation)	Induces tumor regression in murine orthotopic bladder cancer models	‐ (Alternative to BCG)	Preclinical	[190, 191]
	*Lactobacillus rhamnosus* GG (oral)	Enhances the efficacy of anti‐PD‐1 therapy by inducing a cGAS/STING‐dependent type I interferon response in murine models of colorectal cancer and melanoma	ICI (Anti‐PD‐1)	Preclinical	[195]
	*Bifidobacterium breve* and *Bifidobacterium longum*	Associated with improved BCG response and prolonged recurrence‐free survival	BCG	Clinical Association	[84]
	*Lactobacillus rhamnosus* Probio‐M9 (oral)	Synergizes with anti‐PD‐1 therapy by restoring antibiotic‐disrupted gut microbiota; linked to increased butyric acid and T cell cross‐priming	ICI (Anti‐PD‐1)	Preclinical	[192, 193]
	*Lactobacillus gallinarum* (oral)	Improves anti‐PD‐1 efficacy by producing indole‐3‐carboxylic acid (ICA), inhibiting the IDO1/Kyn/AhR axis, and suppressing Treg differentiation	ICI (Anti‐PD‐1)	Preclinical	[194]
	*Blautia coccoides* (oral)	Enhances efficacy of bladder cancer immunotherapy by promoting CD8^+^T cell infiltration	ICI (Anti‐PD‐1)	Preclinical	[198]
	*Bifidobacterium bifidum* (oral)	Enhance the efficacy of anti‐PD‐1 through the IFN‐γ signaling pathway in murine colon and lung cancer models	ICI (Anti‐PD‐1)	Preclinical	[196]
	*Faecalibacterium prausnitzii (oral)*	Enhances the efficacy of dual CTLA‐4/PD‐1 blockade via increased CD8 + T cell infiltration and cytokine production in murine colon cancer and melanoma models	ICI (Anti‐PD‐1 and Anti‐CTLA‐4)	Preclinical	[197]
	*Alistipes onderdonkii (oral)*	Enhance the efficacy of anti‐PD‐1 through elevated CD8 T cell infiltration in murine colon cancer models	ICI (Anti‐PD‐1)	Preclinical	[199]
	Mixture of *Lactobacillus casei* Shirota and *Bifidobacterium breve* (oral)	Enhances the antitumor efficacy of gemcitabine and cisplatin in murine bladder cancer models by activating antigen presentation and recruiting cytotoxic T cells.	Chemotherapy (gemcitabine and cisplatin)	Preclinical	[201]
**Microbiome‐Conscious Antibiotic Use**	Avoid unnecessary broad‐spectrum antibiotics	Prevents disruption of gut/urinary microbiome, preserving ICI efficacy and reducing immunosuppression	ICI	Clinical Review	[175, 176, 177, 184]
	Narrow‐spectrum antibiotics/Bacteriophages (Targeting pks + *E. coli*, etc.)	Selective clearance of pathogenic/genotoxin‐producing bacteria, preserving beneficial flora	Prevention/Adjunct Therapy	Future Direction/Conceptual	‐
**Dietary Intervention and FMT**	High‐fiber diet (prebiotics)	Increases Short‐Chain Fatty Acids (SCFAs) production, systemically modulating immunity	ICI	Clinical Association/Ongoing Clinical Trial	[204, 205]
	FMT	Transfers “responder” gut microbial ecosystem to “non‐responders” to reshape the immune microenvironment.	ICI	Clinical Trial	[206]
**Targeting Microbial Metabolism**	Inhibiting host tryptophan metabolism towards the kynurenine pathway (IDO1 inhibitors)	Relieves T‐cell suppression in the tumor microenvironment and enhances anti‐tumor immunity	ICI, Universal	Clinical trials (some Phase III trials failed to meet endpoints; strategies under optimization)	[207, 208]
	Supplementing beneficial microbial metabolites (e.g., IAA, specific SCFAs, Inosine)	Directly activates anti‐tumor immune pathways (e.g., AhR‐FASN axis for IAA, A2A receptor for Inosine) or induces cancer cell death (e.g., ferroptosis for IAA)	Universal	Future Direction/Conceptual	[140, 169]

Abbreviations: AhR, aryl hydrocarbon receptor; BCG, Bacillus Calmette‐Guérin; cGAS, cyclic GMP‐AMP synthase; CTLA‐4, cytotoxic T‐lymphocyte‐associated antigen 4; FMT, fecal microbiota transplantation; ICI, immune checkpoint inhibitor; ICA, indole‐3‐carboxylic acid; IDO1, indoleamine 2,3‐dioxygenase 1; IAA, indole‐3‐acetic acid; Kyn, kynurenine; LBPs, live biotherapeutic products; PD‐1, programmed cell death protein 1; STING, stimulator of interferon genes; SCFAs, short‐chain fatty acids; Treg, regulatory T cell.

### 4.4 Implementing personalized microbiome‐based strategies in BCa care

The inherent inter‐individual variability of the human microbiome offers a compelling avenue for advancing personalized medicine in bladder cancer care. While currently limited by methodological heterogeneity, integrating detailed microbial profiles into clinical decision‐making contingent upon the establishment of standardized protocols offers the potential to facilitate a critical shift toward precision oncology. This paradigm begins with refined risk stratification at diagnosis, where a patient's specific urinary microbial signature may serve as a non‐invasive prognostic tool (Figure 4A). For instance, the detection of a high‐diversity microbiome enriched in genera such as *Pseudomonas* and *Cupriavidus* has been identified as a marker for high recurrence risk, suggesting that patients harboring these signatures may warrant intensified surveillance schedules and early adjunctive therapies to mitigate progression [71, 79, 80].

**FIGURE 4:**
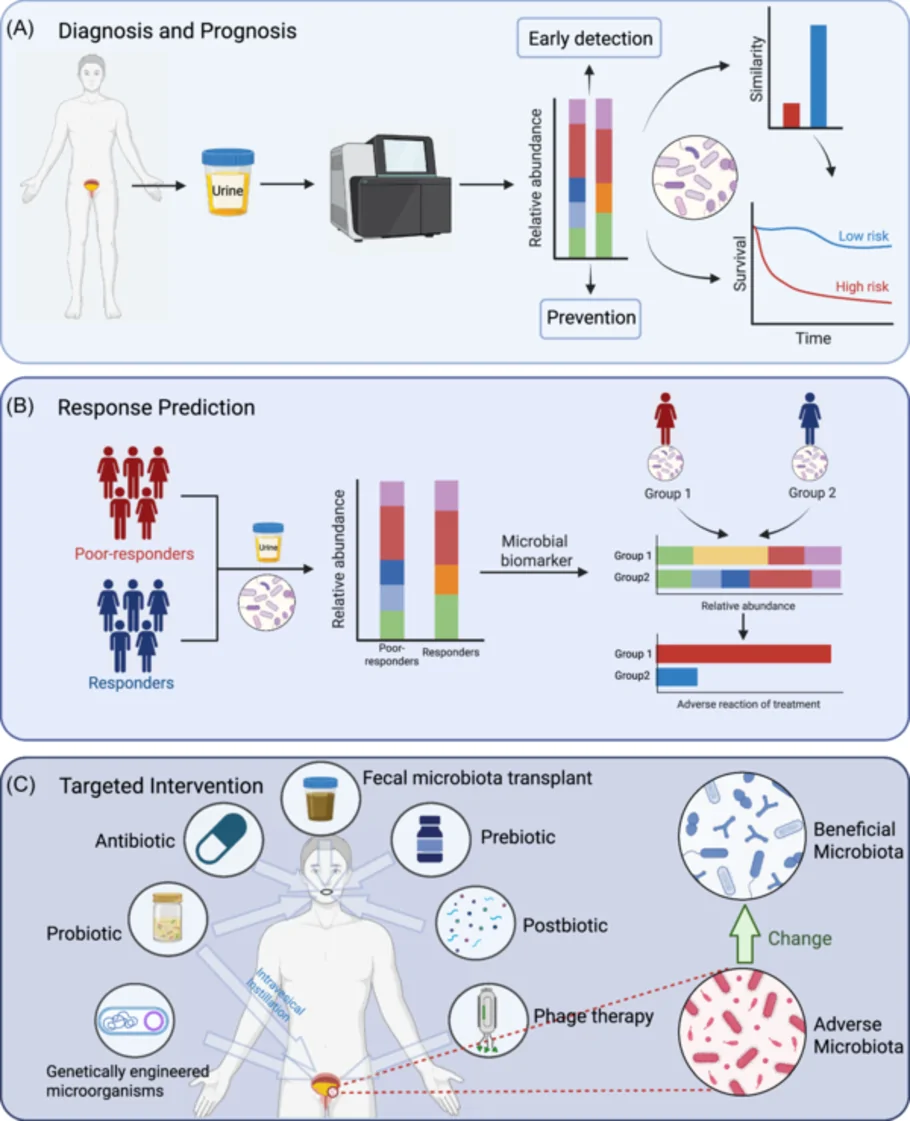
Clinical applications of the microbiome in bladder cancer management. (A) Diagnosis and Prognosis: Schematic workflow showing how urine microbial profiling can identify biomarkers. The graphs on the right represent the potential to stratify patients into high‐risk (red line) and low‐risk (blue line) survival groups based on microbial diversity or specific taxon abundance. (B) Response Prediction: Comparison of microbial signatures between responders and poor responders to immunotherapy. Identification of predictive biomarkers allows for patient stratification prior to treatment. (C) Targeted Intervention: Therapeutic strategies aiming to restore eubiosis include the administration of probiotics, prebiotics, fecal microbiota transplantation (FMT), and bacteriophages. These interventions aim to shift the ecosystem from a dysbiotic, pro‐tumorigenic state to a beneficial state that enhances therapeutic efficacy.

Beyond risk assessment, microbial profiling serves as a potent instrument for guiding therapeutic selection, ensuring that patients are matched with the treatments most likely to succeed. Pretreatment analysis can predict responses to various modalities, directing clinical decision‐making toward effective interventions while avoiding futile toxicity (Figure 4B). Specifically, urobiomes enriched in beneficial taxa such as *Lactobacillus* and *Bifidobacterium* may predict suitability for BCG immunotherapy [84, 131], whereas a gut microbiome dominated by *R. bromii* could signal a poor response to anti‐PD‐1 therapy, prompting the consideration of alternative systemic options [168]. This predictive capability also supports the design of personalized adjunct regimens (Figure 4C), such as the targeted administration of *Lactobacillus*‐based biotherapeutics to patients found to be deficient in these critical taxa prior to BCG instillation.

Furthermore, personalized microbiome management is crucial for optimizing supportive care, particularly regarding antibiotic stewardship. Given the strong negative association between antibiotic use and the efficacy of immune checkpoint inhibitors, the necessity of antibiotic administration must be rigorously reassessed in immunotherapy candidates [175, 176, 177]. When antimicrobial treatment is unavoidable, agent selection should be personalized to minimize disruption to the specific beneficial taxa identified in the patient's microbiome, thereby preserving antitumor immunity. Ultimately, the future of precision oncology in BCa lies in the holistic integration of microbial data with genomic, transcriptomic, and clinical parameters. Such comprehensive diagnostic and prognostic platforms will facilitate the development of management strategies that are inextricably optimized for each patient's unique microbial and genetic landscape.

## 5 METHODOLOGICAL CHALLENGES AND RESEARCH GAPS

### 5.1 Issues with study heterogeneity and small sample sizes

Current investigations into the urobiome face considerable limitations due to pronounced heterogeneity across studies and generally inadequate sample sizes. The lack of standardized protocols for urine collection, preservation, and storage further impedes meaningful cross‐study comparisons. Major sources of variation include differences in participant demographics (e.g., age, sex, hormonal status, and underlying health conditions), urine collection techniques (CC, TUC, or SPA), DNA extraction protocols, sequencing platforms (16S rRNA gene amplicon sequencing vs. WGMS), and bioinformatic analysis pipelines.

This methodological inconsistency directly undermines the interpretability of the clinical associations discussed in earlier sections. A prime example lies in the conflicting reports regarding microbial diversity in BCa. While some studies employing aseptic catheterization report reduced richness in BCa patients, others utilizing voided urine observe significantly higher diversity. These discrepancies should be viewed through a methodological lens: voided urine samples inevitably capture “urogenital” contamination from the distal urethra and genital tract, thereby artificially inflating diversity metrics and obscuring the true low‐biomass profile of the bladder tumor microenvironment. Consequently, high diversity in voided samples may reflect dysbiosis in the external genitalia rather than the bladder itself.

Similarly, the inconsistencies in specific taxonomic biomarkers can be attributed to these sampling biases. Genera such as *Corynebacterium* and *Lactobacillus* are frequently identified as potential markers, yet their enrichment varies wildly across cohorts. Since these bacteria are abundant commensals of the skin and vagina, respectively, their detection in voided urine often represents contamination rather than true bladder colonization. Furthermore, many published urobiome studies are based on small, underpowered cohorts (e.g., *n* < 50), which lack the statistical rigor to control for confounding variables such as sex, age, and antibiotic usage. This lack of statistical power likely explains why certain “promising” markers identified in pilot studies fail to replicate in larger validation cohorts.

Given these challenges, standardization is critical. Evidence indicates that the use of nucleic acid preservatives, shorter storage times, and lower temperatures greatly improves reproducibility of microbial community data [210]. Commercial preservatives such as Assay Assure and Genelock, are increasingly used in clinical urogenital sample collection as it does not introduce more microbial bias [65, 211]. Best practices recommend transferring urine to 4°C within 4 h of collection, with long‐term storage at −80°C [212]. If neither preservatives nor temporary refrigeration are available, the urine should be transferred to −80°C immediately within 3 h after collection [212]. Without rigorous adherence to such standards, distinguishing true biological signals from technical noise remains a formidable challenge.

### 5.2 Standardization of microbiome profiling techniques

Accurate characterization of the urobiome fundamentally depends on the methodologies employed for microbial profiling. Currently, the field lacks a unified, standardized approach, leading to significant challenges in comparing results across studies and in data interpretation. The primary methodological dichotomy lies between culture‐dependent techniques, such as the EQUC, and culture‐independent DNA sequencing technologies, namely 16S rRNA gene amplicon sequencing and WGMS [213].

Although EQUC represents a substantial advancement over standard clinical culture by utilizing varied media, atmospheres (including CO₂ and anaerobic conditions), and extended incubation times to recover a wider array of viable organisms [214, 215], it is inherently limited. It is estimated that even EQUC captures only approximately 72% of the total microbial genera present, missing a significant portion of the urobiome's diversity [216]. This limitation primarily stems from the inability to culture all microorganisms in vitro. Culture‐independent sequencing methods overcome this culture bias but introduce their own set of challenges. A major limitation common to both 16S gene amplicon sequencing and WGMS is their ability to detect DNA from non‐viable, ruptured bacteria, which can lead to misinterpretation of the microbiome's current, active state [58, 217]. To mitigate these complementary limitations, an integrated approach combining EQUC with DNA sequencing is highly recommended, as EQUC confirms bacterial viability while sequencing provides a more comprehensive taxonomic profile [58].

In urobiome research, a major technical challenge is the extremely low microbial biomass in urine, which can lead to inefficient recovery of microbial DNA and result in sequencing data being overwhelmingly dominated by high levels of host (human) DNA. Therefore, the DNA extraction step is particularly critical, aiming to efficiently lyse microbial cells while minimizing co‐extraction of host DNA.

The use of validated commercial kits optimized for urine samples, combined with effective pre‐processing protocols, is crucial for improving DNA quality and result comparability. For example, the Zymo Quick‐DNA Urine Kit is a DNA extraction kit specifically designed for urine. Recent studies have further shown that incorporating a water dilution pre‐treatment protocol when using this kit significantly enhances DNA purity and yields more stable and reproducible extraction results, making it particularly suitable for downstream 16S rRNA gene sequencing analysis [67]. If the research objective involves metagenomic shotgun sequencing, removing host DNA contamination becomes especially important. In such cases, it is recommended to use kits with host depletion functions, such as the QIAamp DNA Microbiome Kit, which employs selective enzymatic steps to degrade host‐derived DNA, thereby actively enriching microbial DNA and increasing the proportion of microbial sequences in the sequencing data.

Regardless of the extraction strategy used, for low‐biomass urine samples, concentrating the sample via centrifugation to increase microbial load, along with strictly including negative controls to monitor potential exogenous contamination introduced during the experimental process, are essential steps to ensure reliable microbial DNA recovery and data credibility.

16S rRNA amplicon sequencing remains the most commonly used method for urobiome taxonomic profiling due to its cost‐effectiveness and high sensitivity for detecting low‐abundance DNA [218]. However, its reliability is significantly compromised by a lack of standardization in several critical areas. Primer selection and hypervariable region bias represent a substantial source of variability, as the choice of which hypervariable region (V1−V9) to amplify dramatically affects results [219, 220]. Different regions exhibit distinct biases, leading to under‐ or over‐representation of specific microbial taxa. For instance, the V3 region tends to underestimate species richness [221]. Although profiling based on the V4 region provides community estimates that align more closely with full‐length 16S sequencing, it is among the least accurate for identifying human‐associated taxa and is prone to co‐amplifying human DNA [56]. The V3 − V4 region is currently the most prevalent choice for 16S rRNA sequencing, offering an optimal balance of technical feasibility, data quality, information content, and community standards, making it the recommended approach for gut microbiota research. For urobiome, however, evidence points to the superiority of the V1−V2 region. As Heidrich et al. demonstrated through their systematic comparison of all nine hypervariable regions in male catheterized urine samples, indicate that V1−V2 provides the highest taxonomic and amplicon sequence variant richness, detects the most exclusive genera, and enables superior species‐level classification with minimal ambiguity [222]. The use of multiple regions (e.g., V1−V6) does not necessarily justify the increased cost and may lead to overestimation of taxonomic richness due to the presence of falsely identified taxa [222].

The selection of read length introduces significant technical challenges in 16S rRNA amplicon sequencing using Illumina's paired‐end 250‐bp chemistry. While longer reads encompassing multiple variable regions (e.g., V1−V3) yield greater information for taxonomic classification, they exhibit progressively deteriorating sequence quality toward their termini. This decline in sequencing quality results in unreliable overlap regions that generate artificial sequences, ultimately inflating diversity measurements through false positive observations [65]. In comparison, shorter target regions (e.g., V4) enable complete overlap between forward and reverse reads, permitting computational correction of sequencing errors through mutual validation of complementary sequences. Accordingly, methodological consensus recommends prioritizing shorter amplicons to minimize technical artifacts and enhance profiling reliability [65].

A fundamental limitation of 16S rRNA amplicon sequencing is that it targets specific genomic regions defined by primers and is subject to inherent primer binding biases, which vary in efficiency across different taxa. Consequently, this method is generally suitable only for taxonomic profiling and measurement of relative abundance [58, 218]. In contrast, WGMS can directly sequencing the DNA of all microorganisms in the sample not only enables the analysis of species composition but also allows for the exploration of functional genes, metabolic pathways. A notable limitation of WGMS, however, is its susceptibility to host‐derived DNA contamination [223]. To compensate for this issue, increased sequencing depth is required to adequately represent the microbial composition. Additionally, when processing low‐biomass or host‐contaminated samples, insufficient sequencing depth limits the ability to predict metabolic pathways for individual taxa, shifting the emphasis toward predictions at the community pathway level [224]. Despite the richer functional insights offered by WGMS, 16S sequencing remains the dominant method in urobiome studies because of the ongoing focus on taxonomic characterization as the primary objective in most investigations.

The standardization of microbiome profiling techniques remains a challenge in urobiome research. While each methodology offers distinct advantages, they are also plagued by significant limitations and biases that hinder cross‐study comparisons and data interpretation. The absence of consensus regarding optimal hypervariable regions for 16S sequencing, combined with the challenges of host contamination in WGMS, underscores the urgent need for standardized protocols and guidelines. Only through such standardization can the field advance significantly. This will enable the generation of reproducible and comparable data, ultimately yielding clinically relevant insights into the urobiome's role in health and disease [37].

### 5.3 Need for longitudinal, mechanistic, and interventional studies

Translating research findings from the human urobiome into clinical insights requires a concerted effort to address critical gaps in our current understanding. To date, the vast majority of urobiome studies have relied on cross‐sectional cohort designs, which are instrumental for comparing individuals with a specific disease state to healthy controls. While these studies are valuable for identifying associations, they are frequently confounded by substantial inter‐individual variability and are inherently limited in their ability to capture temporal dynamics and establish causality.

The temporal stability of a microbial community is a fundamental ecological characteristic and is integral to defining states of dysbiosis [225]. Evidence from other microbiomes suggests that a significant portion of the observed variability can be attributed to temporal fluctuations [226, 227, 228, 229]. Therefore, there is a pressing need for longitudinal studies in which participants provide urine samples over a well‐defined time course. Such designs are crucial for establishing baseline temporal variation within the urobiome and for elucidating the impact of cyclical host physiological processes (e.g., menstrual cycle, hormonal changes) and episodic events (e.g., antibiotic courses, urinary tract infections) on microbial community structure and stability.

Beyond longitudinal observation, a deeper mechanistic understanding is essential. Future research must move beyond correlative analyses to investigate the functional mechanisms through which specific microbes or community structures influence urinary tract physiology and pathophysiology. This necessitates the integration of multi‐omics approaches (e.g., metatranscriptomics, metabolomics, proteomics) with host response profiling to delineate the functional pathways and host‐microbe interactions that under health and disease.

Finally, to move from association to causation, preclinical and clinical interventional studies are paramount. Currently, there is a paucity of rigorous causal validation studies in BCa compared to other malignancies. Future research should prioritize using germ‐free mouse models engaged with FMT from BCa patients to definitively characterize the tumorigenic or therapeutic potential of specific communities. Additionally, targeted interventions, such as probiotic supplementation, bacteriophage therapy, or dietary modifications, conducted within randomized controlled trial frameworks, are required to experimentally test hypotheses regarding microbial influence on clinical outcomes. These studies will provide the highest level of evidence for whether modulating the urobiome can effectively prevent or treat urological conditions.

In summary, advancing the field hinges on a paradigm shift from descriptive, cross‐sectional studies to a more comprehensive research agenda that prioritizes longitudinal sampling, mechanistic inquiry, and rigorous interventional trials to truly decode the functional role of the urobiome and unlock its therapeutic potential.

## 6 CONCLUSIONS AND FUTURE PERSPECTIVES

### 6.1 Summary of current knowledge

Substantial evidence has now overturned the long‐held dogma of urine sterility, confirming that the urinary tract hosts a diverse microbial community characterized by advanced sequencing and enhanced culture methods. This core urobiome is influenced by host factors such as sex, age, and antibiotic exposure. In BCa, distinct microbial ecosystems differentiate patients from healthy individuals, show consistent dysbiosis patterns, which are enrichment of genera like *Enterobacter* and *Acinetobacter*, alongside depletion of protective taxa such as *Lactobacillus*. Beyond mere association, mechanistic studies reveal critical microbiota contributions to carcinogenesis through the induction of chronic inflammation, production of genotoxins, immune modulation, and direct activation of tumor‐promoting pathways. Clinically, microbial diversity and specific signatures show remarkable potential as biomarkers for diagnosis and recurrence prediction, with certain pretreatment profiles strongly correlating with patient outcomes. Moreover, the composition of urinary and gut microbiota significantly influences therapeutic efficacy, particularly for BCG immunotherapy and ICI. Enrichment of *Lactobacillus* in BCG responders and the documented negative impact of broad‐spectrum antibiotics on ICI outcomes underscore the microbiome's central role as a treatment modulator. However, the transition from discovery to routine clinical implementation remains contingent on overcoming the profound inter‐study heterogeneity and lack of standardized protocols identified throughout this review.

### 6.2 Integration of microbiome research into bladder cancer care

Translating microbiome research into clinical practice represents a fundamental paradigm shift in BCa management, promising enhanced diagnostic precision, therapeutic efficacy, and improved long‐term outcomes. Integration will begin with diagnostic innovation, where standardized, non‐invasive urine‐based microbial tests complement existing tools like cystoscopy and cytology. These tests can provide early detection and risk stratification based on identified dysbiotic patterns, particularly for high‐risk populations or in ambiguous clinical cases.

Therapeutically, this translation is already moving from bench to bedside, as evidenced by emerging clinical trials targeting the gut‐bladder axis. For example, regarding antibiotic stewardship, the ongoing REINFORCE trial (NCT06709196) is evaluating whether targeted antibiotic prophylaxis based on preoperative urine culture can reduce postoperative infections compared to standard empiric regimens, a strategy that could minimize microbiome disruption. In the realm of dietary modulation, the DIET study (NCT04645680) is investigating the causal impact of high‐fiber interventions on the gut microbiome to potentially enhance immune checkpoint inhibitor responses in genitourinary cancers. Furthermore, while bladder‐specific FMT trials are nascent, basket trials such as MITRIC (NCT05286294) are currently recruiting patients with genitourinary malignancies to assess if fecal transplants from responders can overcome resistance to PD‐1/PD‐L1 blockade. Ultimately, this integration will enable truly personalized treatment algorithms that incorporate microbial data alongside traditional genomic and clinical parameters.

### 6.3 Call for multidisciplinary collaborations and translational research

Realizing the full clinical potential of microbiome science in BCa care requires concerted efforts across disciplines. The complexity of the host‐microbe‐tumor axis demands collaboration among urology, oncology, microbiology, immunology, bioinformatics, and biomedical engineering.

To ensure the success of future translational efforts, the field must prioritize specific research directions. First, the implementation of contamination‐aware low‐biomass workflows is critical; future studies must strictly standardize sampling to distinguish the bladder mucosal niche from the urogenital environment. Second, longitudinal multi‐center cohorts are essential to capture temporal dynamics and definitively differentiate causal microbial drivers from passenger effects. Finally, moving from correlation to causation requires robust causal inference frameworks and the functional validation of candidate taxa. This can be achieved by integrating multi‐omics (metabolomics, metatranscriptomics) with host response profiling to elucidate the functional pathways driving disease progression. Through these coordinated efforts, microbiome science can deliver tangible advances that improve the lives of BCa patients worldwide.

## AI STATEMENT

During the preparation of this work, the authors used Gemini in order to improve language of the manuscript. After using this tool, the authors reviewed and edited the content as needed and take full responsibility for the content of the publication.

## AUTHOR CONTRIBUTIONS

**Haoxiang Xu** and **Zhibiao Li**: Conceptualization; investigation; literature review; writing—original draft; visualization. **Lin Yang, Jun Zheng, Weijia Li, Rulin Liao, Wenxue Huang, Boyuan Sun, Zhuohang Li, Cunzhen Ma**, and **Xunguo Yang**: Investigation; literature review; data curation; validation. **Zongwei Wang**: Supervision; resources; writing—review & editing. **Jie Zhao, Bisheng Cheng**, and **Peng Wu**: Conceptualization; project administration; funding acquisition; supervision; writing— review & editing. All authors have read the final manuscript and approved it for publication.

## CONFLICT OF INTEREST STATEMENT

The authors declare no conflicts of interest.

## ETHICS STATEMENT

No animals or humans were involved in this study.

## Data Availability

No new data were generated or analyzed in this review. Supplementary materials (graphical abstract, slides, videos, Chinese translated version, and update materials) may be found in the online DOI or iMetaOmics Science http://www.imeta.science/imetaomics/.
